# Supplementary material for: Complement is activated by elevated IgG3 hexameric platforms and deposits C4b onto distinct antibody domains
Source: Nat Commun. 2023 Jul 7;14:4027. doi: 10.1038/s41467-023-39788-5 (PMC10328927; doi:10.1038/s41467-023-39788-5)
Supplement: Supplementary file 1 — Supplementary Information [file 41467_2023_39788_MOESM1_ESM.pdf]

Supplementary Information

Complement is activated by elevated IgG3 hexameric platforms and deposits C4b onto distinct antibody domains

Leoni Abendstein[1], Douwe J. Dijkstra[2], Rayman T. N. Tjokrodirijo[3], Peter A. van Veelen[3], Leendert A. Trouw[2], Paul J. Hensbergen[3] & Thomas H. Sharp[1,*]

[1]*Department of Cell and Chemical Biology, Leiden University Medical Center, 2300 RC Leiden, The Netherlands*

[2]*Department of Immunology, Leiden University Medical Center, 2333 ZA, Leiden, The Netherlands*

[3]*Center for Proteomics and Metabolomics, Leiden University Medical Center, 2333 ZA, Leiden, The Netherlands*

**To whom correspondence should be addressed: t.sharp@lumc.nl*

SUPPLEMENTARY MATERIAL

Supplementary Methods

Supplementary Fig. 1. Overview IgG1, IgG3 and classical complement pathway.

Supplementary Fig. 2. Purification and characterisation of IgG1 and IgG3.

Supplementary Fig. 3. MS analysis of *N*-linked glycans on IgG1 and IgG3.

Supplementary Fig. 4. Tomographic slices of IgG1 and IgG3 bound to antigenic liposome surfaces.

Supplementary Fig. 5. Analysis of ordered Fab domains.

Supplementary Fig. 6. Structures of Fab-Fab interactions that may contribute to array formation

Supplementary Fig. 7. The formation of elevated IgG3 hexamers is driven by associating Fab domains.

Supplementary Fig. 8. Tomographic slices of IgG1 and IgG3 bound to antigenic liposome surfaces and incubated with NHS at 4 °C.

Supplementary Fig. 9. Tomographic slices of IgG3 and IgG1 bound to antigenic liposome surfaces and incubated with NHS at ambient temperature.

Supplementary Fig. 10. Subtomogram averaging routine of IgG3-C1 complexes and FSC curves.

Supplementary Fig. 11. Analysis of IgG3-C1 maps and models.

Supplementary Fig. 12. MS/MS analysis of thioester mediated C4b-IgG3 hinge crosslinked peptides.

Supplementary Fig. 13. MS/MS analysis of thioester mediated C4b-IgG3 Fab crosslinked peptides.

Supplementary Fig. 14. MS/MS analysis of thioester mediated C4b-IgG1 and IgG3 Fc crosslinked peptides.

Supplementary Fig. 15. MS/MS analysis of a tryptic *O*-glycopeptide from IgG3.

Supplementary Fig. 16. MS characterisation of C4b binding to IgG3 compared to negative controls.

Supplementary Fig. 17. Deposited structures and PDB codes used for the model building of gC1q.

Supplementary Fig. 18. Deposited structures and PDB codes used for model building and alignment of C4b.

Supplementary Table 1. BLAST comparison of IgG1 and IgG3 constant heavy chain sequences.

Supplementary Table 2. Lengths and sequences of IgG1 and IgG3 hinge regions.

Supplementary Table 3. Parameters for cryoEM data collection and analysis.

Supplementary References

SUPPLEMENTARY METHODS

Subtomogram averaging of IgG3 Fc platform

All particles used in this section are deposited in the Electron Microscopy Public Image Archive (EMPIAR) with accession code EMPIAR-11406. For the initial template, which was used during the reconstruction of the IgG3 Fab and Fc regions, a bilayer membrane model was used. This model was filtered to 40 Å and white noise was added in EMAN2 using the command e2proc3d.py. Further reconstructions were performed in Dynamo (version 1.1.157) run in MATLAB (version R2021a) [1] (Supplementary Fig. 7a). In Dynamo, particles were binned by 2 and aligned over 4 iterations to the initial model, allowing particles to fully rotate around the z and x axes with sampling discretisation of 30°, a refinement of 5 and refine factor of 2. For this first step, to align all manually picked particles to the surface, no azimuth rotation was allowed, and the shift limitations were set to 12 pixels in each direction from the centre of the particle box. Other than these parameters, the default values in Dynamo were used for global refinement. Next, the alignment of the particles was refined by also allowing the particles to azimuthally rotate over a range of 360° with sampling every 30°, a refinement of 5, refine factor of 2, and rotation around the z and x axes over a range of 20° sampling every 10°. Afterwards, multiple iterations with rotation around the z and x axes over a range of 5° with sampling every 2°, and decreasing azimuthal rotation ranges from 360° with sampling every 30° to 30° with sampling every 5°, to align particles precisely to the lipid membrane. For all these steps, the refine was set to 5 with a refinement factor of 2. Next, the 40 Å lowpass-filtered initial model was again used as a template and the previously estimated positions were used for final alignment, letting the particles rotate fully around their x and z axes with a sampling discretisation of 30° for 6 iterations. This step was calculated with refine of 5 and a refine factor of 2. Afterwards, rotations around the x and z axes were skipped, but the azimuthal rotation was set to 360° with a sampling of 30° for 6 iterations, followed by 6 iterations with azimuthal rotation of 180° with sampling every 20° and another 6 iterations with azimuthal rotation of 30° with sampling every 5°. For all iterations, refine was set to 5 with a refine factor of 2. These steps were followed by a final alignment round of 3 iterations with a rotation range around the x and z axes set to 45° and sampling every 5°, as well as an azimuth rotation range of 30° with sampling every 5°, with the refinement set to 4 and a refine factor of 2 and shift limits set to 30 pixels. The resulting average yielded the overall IgG3 map, which was dominated by the Fab-membrane density (top orange red map; Supplementary Fig. 7a).

To refine the Fc domain, we generated a smaller spherical mask centred on the Fc region with a radius of 40 pixels and a Gaussian filter of 5 in Dynamo, which was used for classification. The previously estimated particle positions were used during multi-reference alignment to limit the membrane dominating classification. The final average from the previous refinement was copied 6 times and noise with an amplitude of 5 was added in Dynamo. The classification was started by letting the particles rotate 45° around their x and z axes with sampling every 5°, azimuthal

rotation of 30° with sampling every 5°, and refinement set to 5 with refine factor of 2 for 3 iterations. For the following iterations, rotations around the x and z axes were skipped and particles were allowed to azimuthal rotate within 10° with sampling every 2°, refine was set to 4 and the refine factor to 2 for 3 iterations, followed by another 3 iterations with an azimuthal rotation range of 6° with a sampling of 1°, refine of 3 with a refine factor of 2. This classification was finalised with 3 additional iterations, with an azimuthal rotation range of 2° and sampling every 0.5° and refinement of 2 with refine factor set to 2. Shift limitations were also decreased from 30 to 2 pixels in all directions over 12 iterations, yielding the classes shown in Supplementary Fig. 7a.

Multi-reference alignment resulted in a class presenting a clear Fc platform containing 571 particles (dark cyan map; Supplementary Fig. 7a). This class was used for further refinement. For the final average of the Fc region, the particles were divided into 2 half datasets, even and odd, and two independent alignments were performed using the previously calculated average as a template and the estimated particle positions and orientations. A small elliptical mask centred on the Fc region with 50 pixels in x and y, and 30 pixels in z, with a Gaussian filter of 5 was generated in Dynamo. For both datasets, 30 iterations were performed. During the first 6 iterations, the particles were allowed to rotate around their x and z axes within a range of 10° with sampling every 2° to limit the Fc platform rotating away from the membrane, which was outside the mask. The azimuth rotation range was set to 360° with sampling every 45°, with the refine set to 5 with refine factor of 2. The shift limits were set to 4 × 4 × 8 pixels in x, y and z. For the next 12 iterations, rotations around the x and z axes were skipped and only azimuthal rotations were calculated. Initially, rotations were set to 180° with sampling every 30° with a refine of 4 and refine factor set to 2 for 10 rounds. The shift limits were set to 4 pixels in all directions. Next, the rotations were set to 30° with sampling every 10° and refine set to 4 with the refine factor set to 2. Shift limitations were set to 2 pixels per direction. For the following 6 iterations, particles were free to rotate in a range of 10° with sampling every 2° and refine set to 4 with refine factor set to 2 and shift limits set to 1 pixel. The final 6 iterations used a rotation range of 2° with sampling every 0.5° and refine set to 4 with refine factor set to 2. The shift limits were again set to 1 pixel per direction. The resulting averages were further used to calculate the Fourier shell correlation (FSC) curves in EMAN2 (Supplementary Fig. 7b), as described in the main text, which reached 19 Å (dark blue map). All the previously described subtomogram averaging steps in Dynamo were done without applying symmetry. However, there was clear C6 symmetry in the final average (Supplementary Fig. 7c), and so the above was repeated on the 571 even/odd half-datasets but with C6 symmetry applied. For this C6-symmetrised map, the FSC reached 14 Å (magenta map; Supplementary Fig. 7).

Classification and subtomogram averaging of IgG3-C1-C4b

All particles used in this section are deposited in EMPIAR with accession code EMPIAR-11407. The initial model of the IgG3-C1-C4b map described in the main Methods section was used for

subtomogram averaging in Dynamo (Supplementary Fig. 10b), where, if not mentioned differently, default values were used. Particles were binned by 2 and aligned to this model over 4 iterations, allowing particles to fully rotate around the z and x axes with sampling discretisation of 30°. No azimuthal rotation was allowed. Refine was set to 5 with a refine factor of 2. Shift parameters were set to 12 pixels in each direction from the centre of the particle box, and a spherical mask with a radius of 110 pixels, generated in Dynamo, was used. The resulting average was used as a template and the estimated particle coordinates as a table for the following alignment. This alignment was split into 5 rounds and a spherical mask with a radius of 78 pixels was used. The first round contained 3 iterations and particles were binned by 4. The rotation range around the particles' x and z axes as well as the azimuth rotation range were set to 360° with sampling every 60° and refine was set to 5 with refine factor of 2. Shift limits for this round were set to 12 pixels in each direction. The second round contained another 3 iterations and particles were binned by 2. Now, particles were allowed to rotate within a range of 20° with sampling every 5° around their x and z axes. The azimuth rotation range was set to 60° with sampling every 5° and the refine was set to 2 with a refine factor of 2. The shift limits for this round were set to 4 pixels in each direction. The next round contained 3 iterations and the particles were again binned by 2. This time, the rotation around the x and z particle axes and the azimuth rotation range was set to 4° with a sampling of 2° and the refine set to 2 with refine factor set to 2. The shift limits were set to 2 pixels per direction using particles binned by 2. The fourth round contained 5 iterations with rotation ranges for their x and z axes and the azimuthal rotation range set to 4° with sampling every 2°, and the refine set to 3 with refine factor set to 2. For these iterations, the particles were binned by 2, the shift limits were set to 2 pixels in each direction. For the last round in this refinement, unbinned particles were used. The rotation range for rotating the particle around their x and z axes as well as the azimuth rotation range was set to 4° with sampling every 2° and refine set to 3 with the refine factor set to 3. The shift limits were set to 2 pixels in each direction.

Next, multireference classification was performed over 4 rounds by cloning the resulting average 3 times and adding noise with an amplitude of 3 in Dynamo. A spherical mask with a radius of 110 pixels was used for this classification. During the first 3 iterations, particles were binned by 4 and allowed to rotate within a range of 20° around their x, z and azimuthal axes, with a sampling of 5°. Refine was set to 5 with the refine factor of 2 and shift limits were set to 4 pixels in each direction. For the next 3 iterations, particles were binned by 4 and the rotation around the x and z axes of the particles and the azimuth rotation range were set to 4° with sampling every 2°. Refine was set to 2 with a refine factor of 2, and the shift limits during this round were set to 2 pixels per direction. For the next 5 iterations, particles were binned by 2 and allowed to rotate around their x and z axes and to azimuthally rotate within a range of 4° with sampling every 2°, with a refine set to 3 with a refine factor of 2. The shift limitations were set to 2 pixels in each direction. For the final 5 iterations in this multireference refinement, the particles were used unbinned. The rotation range around the particles' x and z axes as well as the azimuth rotation range were set to 4° with sampling every 2°. Refine was set to 3 with a refine factor set to 1.

Shift limits were set to 1 pixel per direction. This classification resulted in 2 classes with clear C1 complexes bound to antibodies on a membrane, which contained 1,612 and 816 particles (pastel lime and pastel blue maps, respectively; Supplementary Fig. 10b).

The two classes resulting from multireference refinement were combined, and the remaining 2,428 particles were used for all following refinements (Supplementary Fig. 10b). The dataset was split into 2 half datasets, even and odd, before the 2 independent alignments were performed. The initial model from the beginning was used, together with a spherical mask of radius 110 pixels. For the even and odd refinements, 5 rounds, each containing 6 iterations were performed. For the first round, particles binned by 2 were used and were allowed to rotate azimuthally within a range of 180°, sampling every 30°. The refine was set to 5 with a refine factor of 2. Shift limits were set to 4 pixels per direction. For the second round, particles were again binned by 2 and the azimuth rotation range was set to 30° with sampling every 10° and refine set to 4 with a refine factor of 2, and shift limits were set to 2 pixels per direction. During the third round, particles binned by 2 were allowed to rotate azimuthally within a range of 10° and sampling every 2°. The refine was set to 3 and the refine factor set to 2, and the shift was limited to 1 pixel per direction. For the fourth round, unbinned particles were used that were allowed to rotate in an azimuth rotation range of 2° with sampling every 0.5°. Refine was set to 2 with a refine factor of 2, and the shift limits for this as well as for the next round were set to 1 pixel per direction. The fifth round was performed on unbinned particles. The azimuth rotation range was set to 1° with sampling every 0.5° and refine set to 1 with refine factor of 2. All refinements were performed without applying symmetry. This resulted in an overall IgG3-C1-C4b map, which reach 34 Å resolution (sky blue map; model **2**, Supplementary Fig. 10b).

Next, we performed multiple focused refinements to attempt to improve the resolution of different parts of the structure. We used the overall IgG3-C1-C4b map from above as an initial model and the estimated particle positions and orientations. First, we focussed on the C1 complex. We generated a spherical mask with a radius of 65 pixels centred on the C1 complex (Supplementary Fig. 10b). We used all 2,428 particles split into even and odd datasets. These 2 independent refinements were performed over 14 iterations. For the first 2 iterations, particles were binned by 2 and these were allowed to rotate within a range of 30° around their x and z axes with sampling every 5°. The azimuth rotation range was set to 360° with sampling every 45° and refine was set to 5 with refine factor set to 2. The shift limits were set to 4 pixels per direction. For the next 2 iterations, particles were again binned by 2. These particles were then allowed to rotate around their x and z axes within a range of 10 and sampling of 2°. Additionally, these particles were allowed to rotate azimuthally within a range of 180° and sampling of 30°. Refine was set to 5 with a refine factor of 2 and shift limits were set to 4 pixels per direction. The following 4 iterations used particles binned by 2 with a rotation range around the x and z axes and azimuth rotation range both set to 10° with sampling every 2°, and the refine was also set to 2 with refine factor of 2. The shift limit was set to 2 pixels in each direction. For the next 4 iterations, unbinned particles were used, which were allowed to rotate around their x and z axes over a range of 2° with sampling every 0.5°. The azimuth rotation range was set to 2° with

sampling every 0.5°. Refine was set to 1 with the refine factor set to 2 and a shift limit of 1 pixel per direction was allowed. For the final 2 iterations, unbinned particles were allowed to rotate around their x and z axes and azimuthally rotate within a range of 1° with sampling every 0.5°. The refine was set to 1 with a refine factor of 2 and the shift limits were set to 1 pixel per direction. The resulting IgG3-C1-C4b focussed on the C1 region map can be seen in Supplementary Fig. 10b,c (dark green map; model **3**).

To yield insights into how the proteases are oriented within the C1 complex, we generated a mask with a diameter of 46 pixels and a height of 26 pixels centred on the protease domain which included the C1 globular head regions (Supplementary Fig. 10b). We used the previously generated IgG3-C1-C4b focussed on the C1 region map as a template. The particles were again split into even/odd datasets, each dataset containing the same particles as the previous half-datasets. Particles were binned by 2 and over 6 iterations allowed azimuth rotation over a range of 30° with a sampling of 5°, refine was set to 5 with the refine factor set to 2. The shift limits were set to 4 pixels per direction. For the next 6 iterations, particles binned by 2 were free to azimuthally rotate within a range of 10° with a sampling of 2°. Refine was set to 2 with the refine factor set to 2 and the shift limits were set to 2 pixels per direction. For the final 6 iterations, unbinned particles were allowed to azimuthally rotate within a range of 2° with a sampling of 0.5°. Refine was set to 1, the refine factor was set to 2 and the shift limits were set to 1 pixel per direction. This average resulted in the focussed C1 globular head region (Supplementary Fig. 10b; purple map; model **4**).

Next, we focussed on the antibody Fc platform. For this focused refinement, we generated a mask with a radius of 50 pixels centred on the Fc region (Supplementary Fig. 10b). Model **2** was used as an initial model. The same rotation scheme as for the focussed C1 refinement described above was used, but by using the Fc-specific mask. This resulted in the IgG3-C1-C4b focussed on the Fc region map (Supplementary Fig. 10b; dark red map; model **5**).

The resulting average and refined particles were used to focus the refinement further to the Fab-C4b region. Therefore, we generated a cylindrical mask with a radius of 35 pixels and height of 80 pixels with a Gaussian filter of 5 centred on the C4b-Fab region (Supplementary Fig. 10b). The same even and odd datasets from Fc focussed model above were used. For the first 5 iterations, we used binned by 2 particles, that were free to rotate around their x and z axes and azimuthally rotate within a range of 30° with a sampling of 10°. Refine was set to 5 with a refine factor of 2 and the shift limits were set to 4 pixels in each direction. The following 5 iterations again used particles binned by 2, which could rotate around their x and z axes within a range of 10° with a sampling of 2°. The azimuth rotation range was set to 10° with sampling every 2°. Refine was set to 5 with a refine factor of 2 and shift limits of 2 pixels per direction. The next 5 iterations also used particles binned by 2. These particles were allowed to rotate around their x and z axes and azimuthally rotate within a range of 2° with a sampling of 0.5°. Refine was set to 2, with a refine factor of 2 and a shift limitation of 2 pixels per direction. For the final 2 iterations, unbinned particles were used. Particles were allowed to rotate around their x and z

axes as well as azimuthally rotate within a range of 1° with sampling every 0.5°. Refine was set to 1 with a refine factor of 2 and shift limits were set to 1 pixel per direction. These rounds of refinement resulted in the IgG3-C1-C4b focussed on the C4b region map (Supplementary Fig. 10b; ochre yellow map; model **6**). The focused refinements around the C1-Fc region, C1rs platform, and Fab-C4b region including the used masks are shown in Supplementary Fig. 10b. FSC for each refinement were calculated using EMAN2 after aligning the two even/odd half-maps and applying a tight mask generated in e2filtertool.py from the EMAN2 suite (Supplementary Fig. 10c).

Modelling the C1 complex

The six C1q globular head domains (gC1q) were fit into model **3** (Supplementary Fig. 10b) as rigid bodies using 1PK6 [2] as a model. The heterotrimeric gC1q domain is approximately spherical, and so known orientation from PDB model 6FCZ [3] was used to orient the domains in the map (Supplementary Fig. 17), such that chains B and C were adjacent to the IgG3-Fc region, as previously shown [3, 4]. The collagen arms of C1q are composed of six heterotrimeric collagen fibrils, which were modelled using ccbuilder 2.0 [5] using default values for radius and pitch. The region of the collagen arms between the $C1r_2s_2$ protease platform and gC1q domains, comprising residues A58-89, B60-91 and C57-88 (A, B and C refer to the individual chain within the heterotrimeric structure), were fit into the map as rigid fibrils between the relevant gC1q domain and their locations in the protease platform, which bind via known lysine residues [6]. Next, the C1q stalk, comprising residues A1-39, B1-41, and C1-38 were fit into the map and known disulfide bonds formed between the C-C and A-B interchain cysteine residues. The C1q stalk and collagen region between the $C1r_2s_2$ platform and gC1q domains are separated by residues A40-57, B42-59 and C39-56, which were oriented in the map to connect these two regions. The complete C1q structure was formed by forming bonds between the termini using ISOLDE within UCSF ChimeraX [7, 8], which were then allowed to relax in a brief simulation within the map to yield the final model of C1q in Fig. 3e. The $C1r_2s_2$ proteases were modelled based on the crystal structure of the CUB1-EGF-CUB2 (Supplementary Fig. 1) heterotetramer with PDB code 6F1C [9]. The C1r protease arms, comprising the CCP1-CCP2-SP domains, were based on PDB model 1GPZ [10]. These were fit into the map as rigid bodies and linked to the relevant CUB2 domains using ISOLDE. The C1s protease arms were modelled based on PDB model 4J1Y [11]. The different orientations of C1s were modelled by rotating the CUB2 domain around the C1q collagen arms as described in the main text. These were also linked together using ISOLDE to form the complete $C1r_2s_2$ heterotetramer. The C1q and $C1r_2s_2$ models were placed in the map together and briefly simulated in ISOLDE to yield the complete C1 complex.

Supplementary Figures

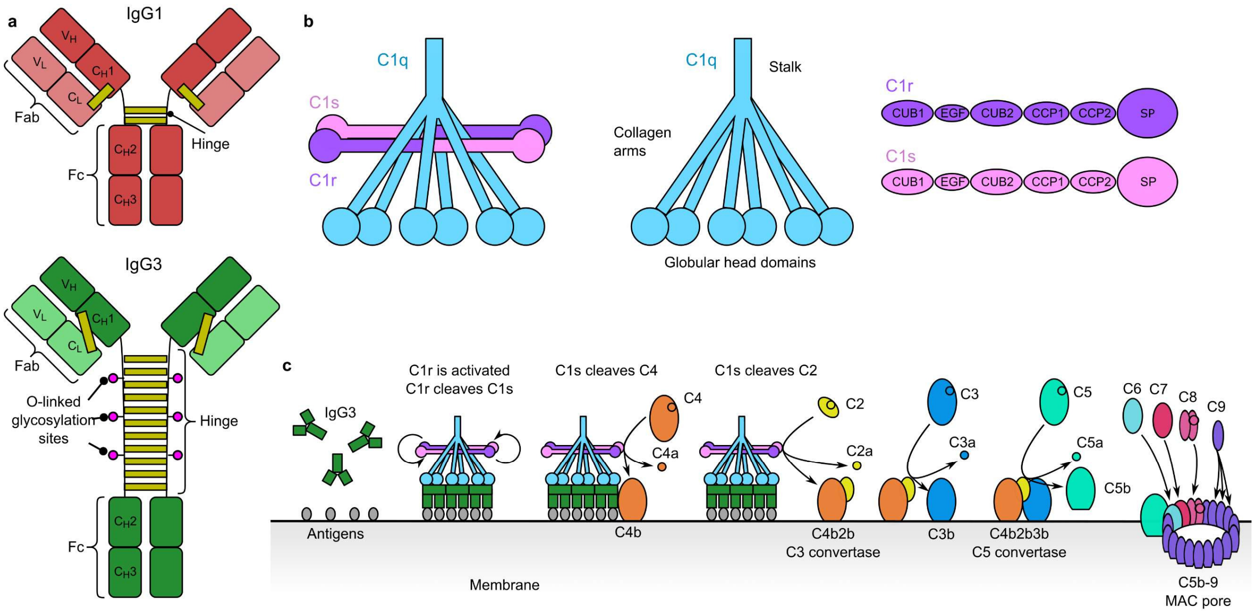

Supplementary Fig. 1. Overview IgG1, IgG3 and classical complement pathway.
(**a**) Schematic showing the domain structures of IgG1 (top, red) and IgG3 (bottom, green). Heavy and light chains are denoted with dark and light colours, respectively. Variable and constant heavy and light chains are denoted with V, C, $_H$ and $_L$, respectively. The fragment of crystallization (Fc), a fragment of antigen binding (Fab) and hinge regions (Hinge) and also labelled, as are the inter-chain disulfide bonds (yellow rectangles) and locations of O-linked glycosylation (magenta spheres). (**b**) Schematic showing the domain structures and names of the components of the C1 complex; C1q (blue), C1r (purple) and C1s (pink). (**c**) Simplified diagram of the classical complement cascade, from C1 activation through to membrane attack complex (MAC) pore formation.

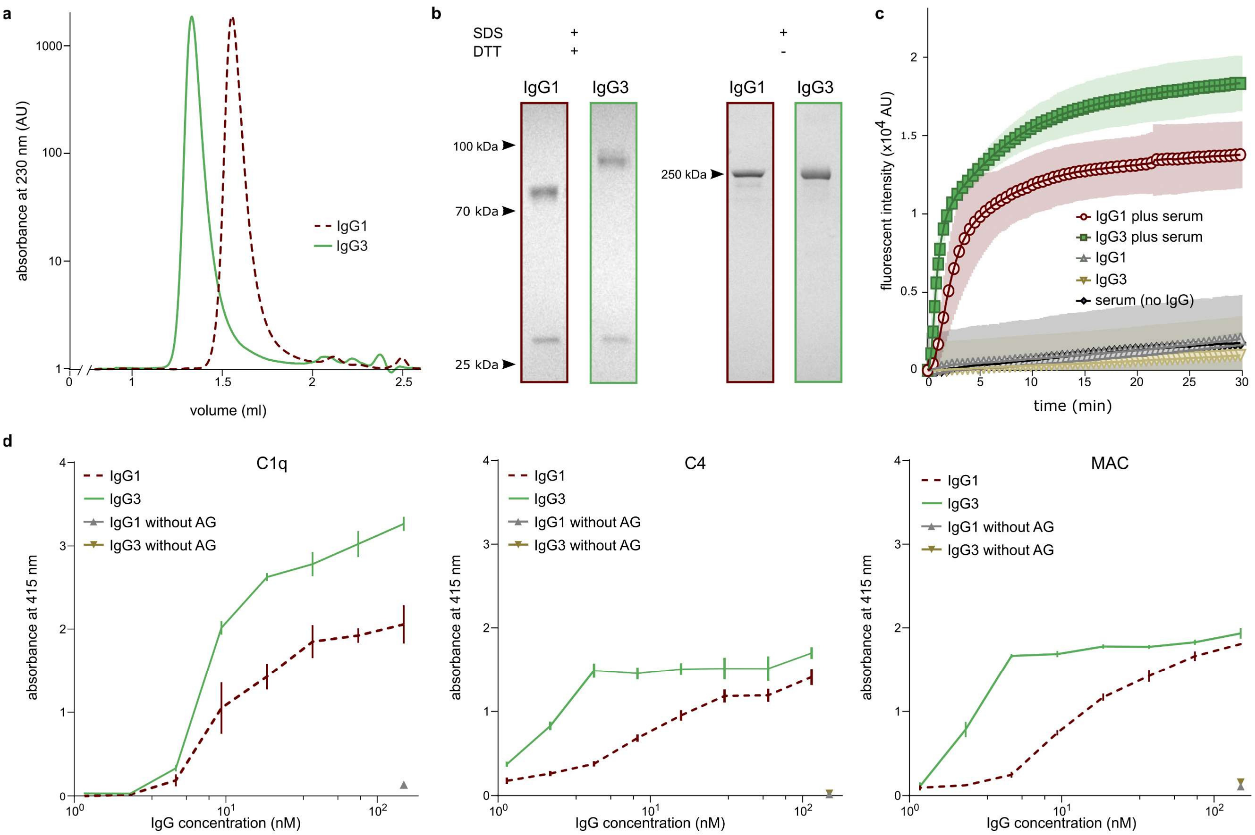

Supplementary Fig. 2. Purification and characterisation of IgG1 and IgG3.
(**a**) Size exclusion chromatography traces of IgG1 (red) and IgG3 (green). (**b**) Coomassie-stained SDS-PAGE of IgG1 and IgG3 in the presence of SDS and/or DDT. Arrowheads indicate standard protein markers of the indicated molecular weight. (**c**) Complement-mediated liposome lysis assays of IgG1 (red) and IgG3 (green) using 50 nM IgG Data are presented as mean values ± SD of 4 independent measurements. (**d**) ELISA showing detection of C1q, C4 and membrane attack complex (MAC; complement protein C5b-C9) binding to antigen-bound IgG1 (red) and IgG3 (green). Antigens (biotin-DNP) were immobilised on streptavidin-coated wells. Data are presented as mean values ± SD of 5 independent measurements containing 3 technical replicates each.

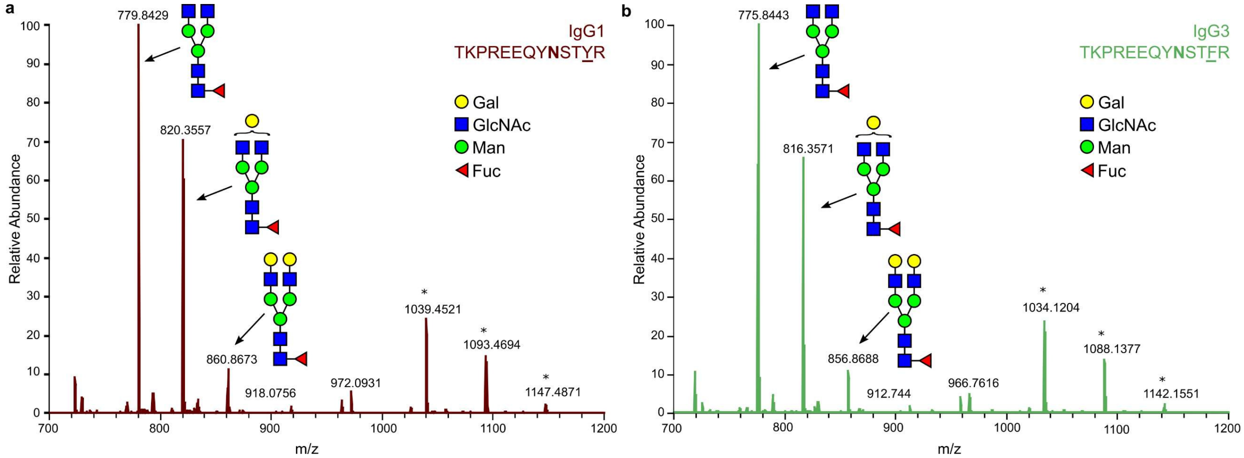

Supplementary Fig. 3. MS analysis of N-linked glycans on IgG1 and IgG3.
N-linked glycans on the (**a**) IgG1 tryptic peptide TKPREEQY**N**STYR (red), and the (**b**) IgG3 tryptic peptide TKPREEQY**N**STFR (green). For both samples, summed MS spectra for the quadruply charged peptide carrying H3N4F1 (m/z 779.8429 and 775.8443 for IgG1 and IgG3, resp.), H4N4F1 (m/z 820.3557 and 816.3571 for IgG1 and IgG3, resp.) and H5N4F1 (m/z m/z 860.8673 and 856.8688 for IgG1 and IgG3, resp.) are depicted. H, hexose; N, N-acetylglucosamine; F, fucose. The 3+ ions of these three species are also visible at higher m/z values indicated with an asterisk. Both antibodies show highly similar glycosylation. Sequences show the site of N-glycosylation (bold) and the single amino acid difference between IgG1 and IgG3 (Y>F; underlined). Gal (Galactose; yellow circle), GlcNAc (N-acetylglucosamine; blue square), Man (mannose; green circle) and Fuc (fucose; red triangle).

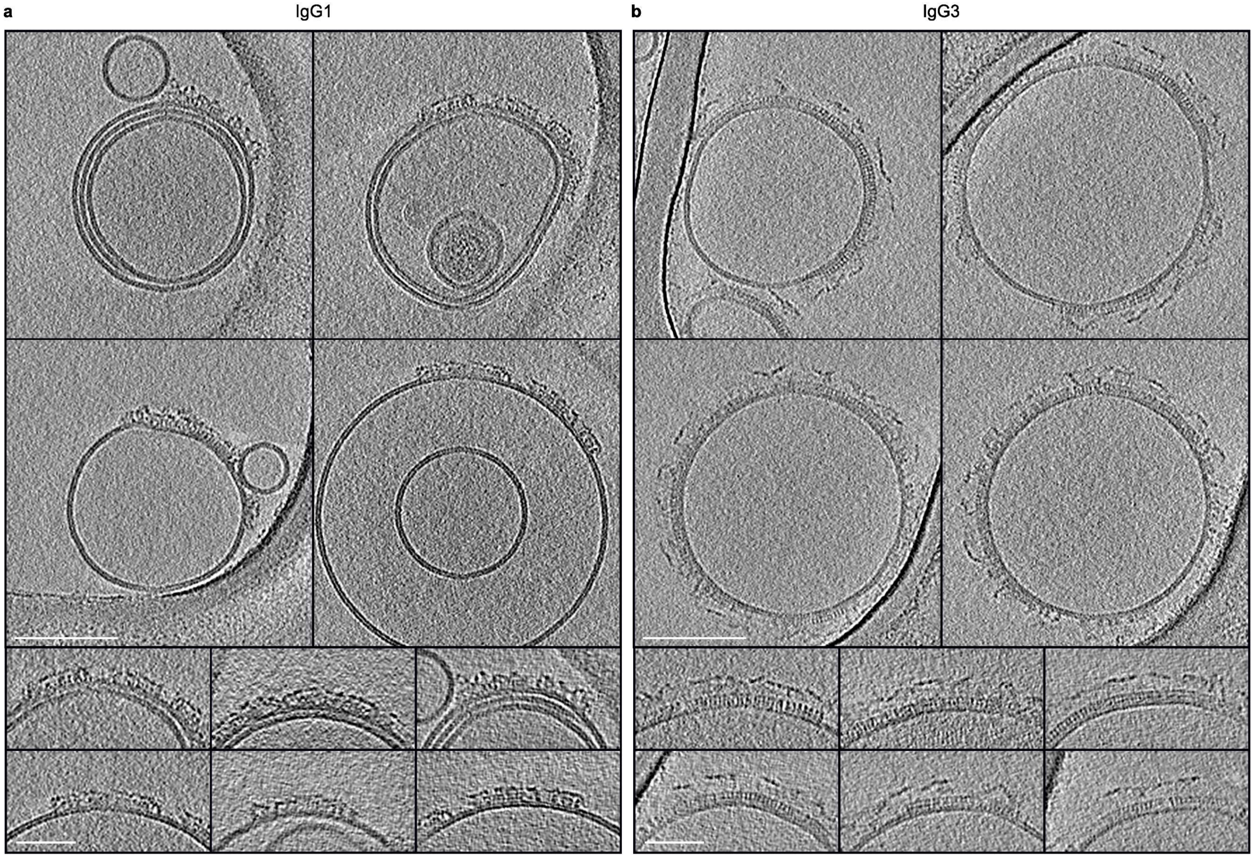

Supplementary Fig. 4. Tomographic slices of IgG1 and IgG3 bound to antigenic liposome surfaces. (**a**) Slices 10 nm thick through tomograms of antigen-bound IgG1. (**b**) Slices 10 nm thick through tomograms of antigen-bound IgG3. Scale bars represent 100 nm (top) and 50 nm (bottom) for both **a** and **b**.

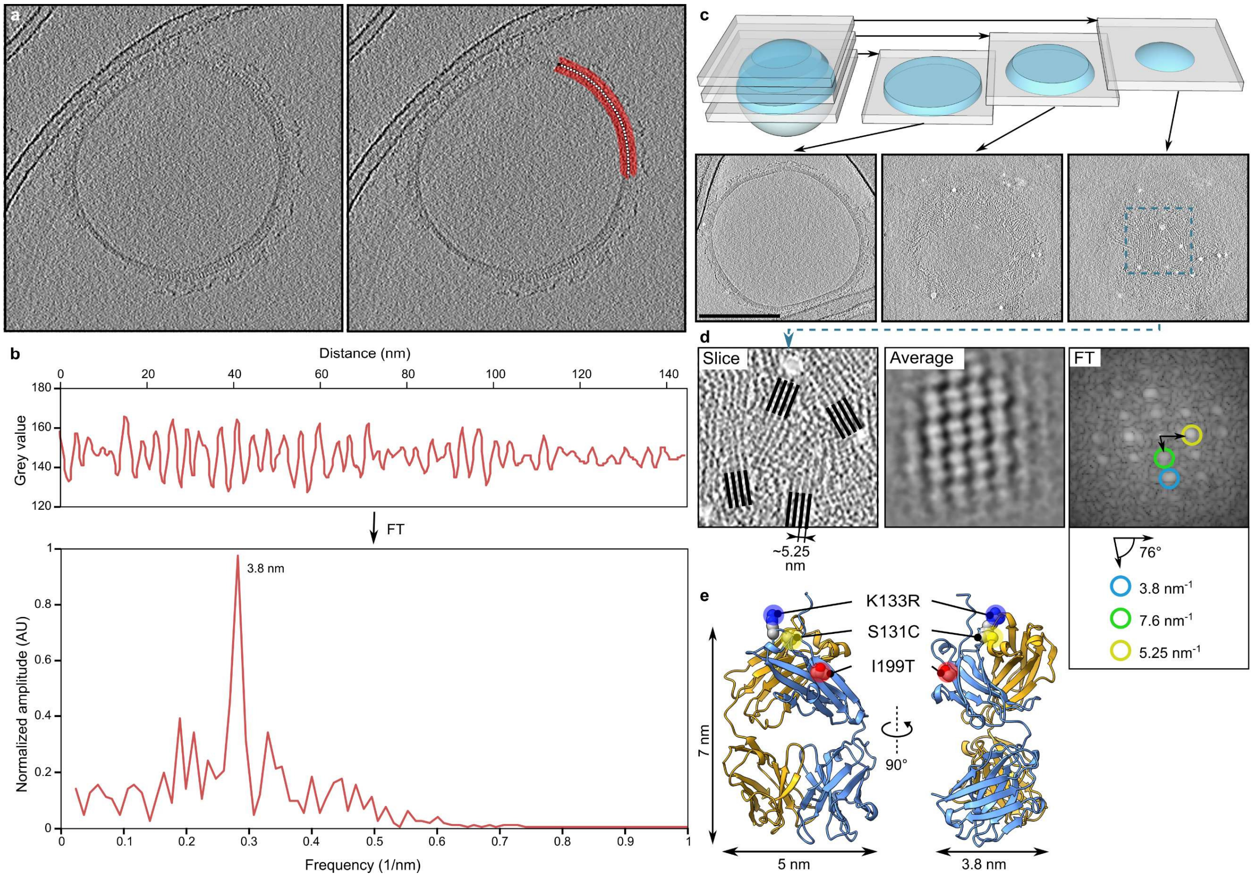

Supplementary Fig. 5. Analysis of ordered Fab domains.
(**a** & **b**) Fourier transforms (FT) reveal the striations caused by aligned Fab domains have a repeating distance of 3.8 nm. (**c**) Schematic showing locations of 7 nm-thick tomographic slices through liposomes shown below. The scale bar represents 200 nm. (**d**) The magnified region shown in the last panel of c and averaged Fab domains with associated Fourier transform. Rings at frequencies 3.8 nm^{-1} and 5.1-5.4 nm^{-1} are highlighted with blue and yellow arcs, respectively. The 7.6 nm peak (green arc) is twice the 3.8 nm distance and corresponds to a pair of Fab domains, each 3.8 nm apart. (**e**) Fab domain dimensions (PDB code 1HZH) with locations of amino acid differences between IgG1 and IgG3 Fab domains highlighted. Heavy and light chains are blue and yellow, respectively.

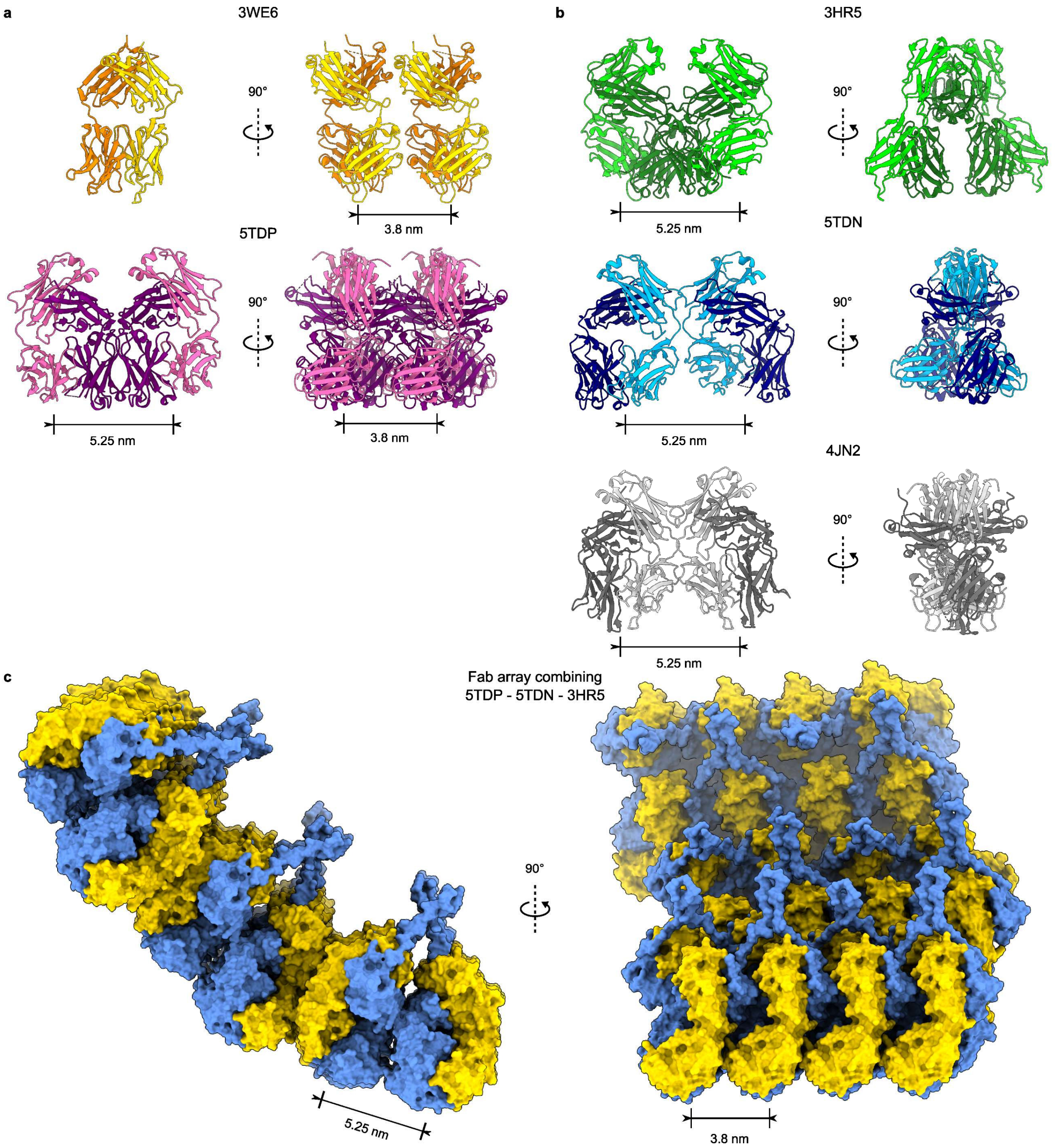

Supplementary Fig. 6. Structures of Fab-Fab interactions that may contribute to array formation. (**a**) Structures with PDB code 3WE6 (crystal structure of anti-Prostaglandin E2 Fab fragment at a resolution of 2.02 Å) and 5TDP (crystal structure of anti-HER2 Fab fragment at a resolution of 1.72 Å), which both have a distance between Fab arms of 3.8 nm. For 5TDP, there is also a repeating distance of 5.25 nm perpendicular to the 3.8 repeating unit. (**b**) Structures with PDB code 3HR5 (humanized 47H4 antibody Fab fragment at a resolution of 2.40 Å), code 5TDN (crystal structure of anti-HER2 Fab fragment at a resolution of 1.63 Å) and code 4JN2 (Fab fragment of anti-Dabigatran at a resolution of 1.71 Å), all have a distance between Fab arms of 5.25 nm. (**c**) Possible combination of 5TDP-5TDN-3HR5 to build a model with the correct distances found in the subtomogram average shown in Fig. 1. Although this model recapitulates the measured distances, this model also presents a pronounced curve away from any antigenic membrane.

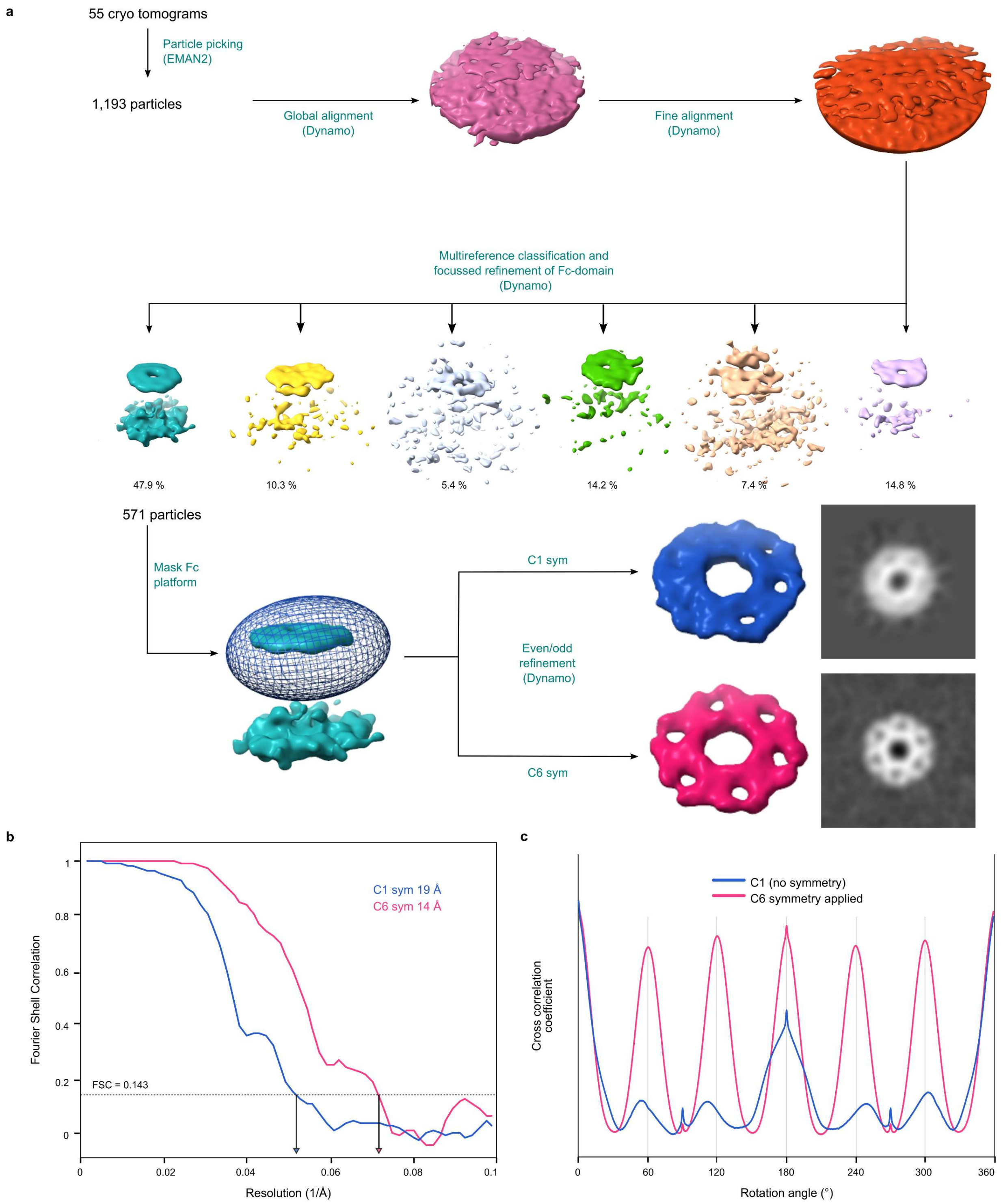

Supplementary Fig. 7. The formation of elevated IgG3 hexamers is driven by associating Fab domains.

(**a**) Simplified subtomogram averaging routine for elevated IgG3-Fc domains. Also shown are slices through the final maps at the IgG-Fc region. (**b**) Fourier shell correlation curves for final Fc-focussed maps with C1 (no symmetry) and C6 symmetry applied. Resolutions reported at FSC = 0.143. (**c**) Rotational correlation plots showing C6 symmetry of the C1(no symmetry applied) and C6-symmetrized maps.

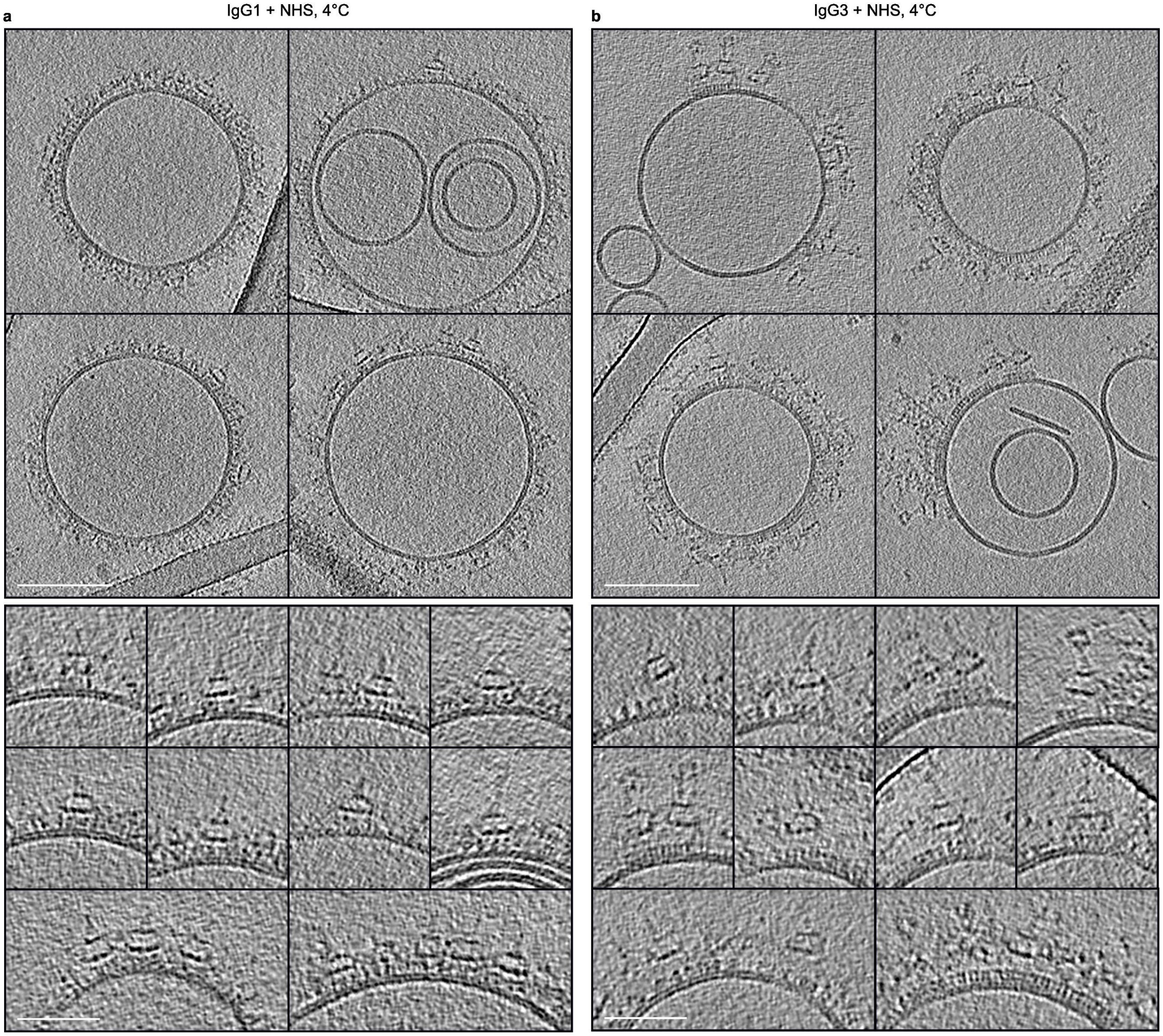

Supplementary Fig. 8. Tomographic slices of IgG1 and IgG3 bound to antigenic liposome surfaces and incubated with NHS at 4 °C.
(**a**) Slices 10 nm thick through tomograms of antigen-bound IgG1 showing whole liposomes (upper 4 panels) and individual IgG1-C1 complexes (lower 10 panels). (**b**) Slices 10 nm thick through tomograms of antigen-bound IgG3 showing whole liposomes (upper 4 panels) and individual IgG3-C1 complexes (lower 10 panels). Scale bars represent 100 nm (top) and 50 nm (bottom) for both **a** and **b**.

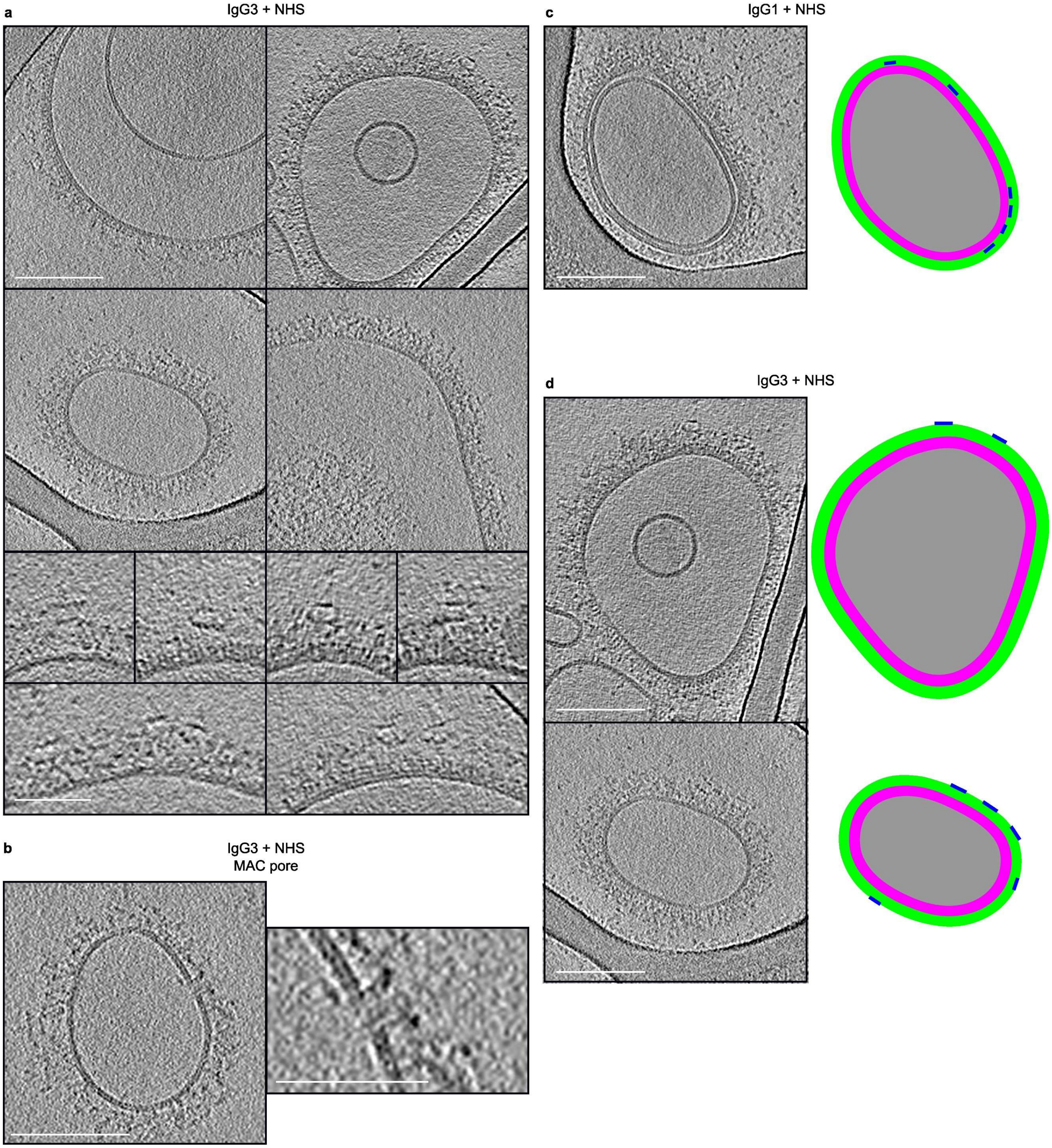

Supplementary Fig. 9. Tomographic slices of IgG3 and IgG1 bound to antigenic liposome surfaces and incubated with NHS at ambient temperature.

(**a**) Slices 10 nm thick through tomograms of antigen-bound IgG3 after complement activation showing opsonized liposomes (upper 4 panels) and individual IgG3-C1 complexes (lower 6 panels). Scale bars represent 100 nm (top) and 50 nm (bottom). (**b**) MAC pore formed after IgG3-mediated complement activation. Scale bars represent 100 nm (left) and 50 nm (right). (**c**) Protein corona on IgG1-bound liposomes after complement activation. A different slice of the denoised version of this tomogram is shown in Fig. 3c. Tomogram slices (left) are annotated (right) with the approximate height of bound proteins. Pink indicates ~11 nm high thick coat, green indicates ~25 nm-high looser coat, and blue lines are IgG1-Fc platforms. The scale bar represents 100 nm. (**d**) Protein corona on IgG3-bound liposomes after complement

activation. A different slice of the denoised version of the tomogram in the lower panel is shown in Fig. 3d. Tomogram slices (left) are annotated (right) with the approximate height of bound proteins. Pink indicates a ~11 nm high thick coat, green indicates a ~25 nm high looser coat and blue lines are IgG3-Fc platforms. Scale bars represent 100 nm.

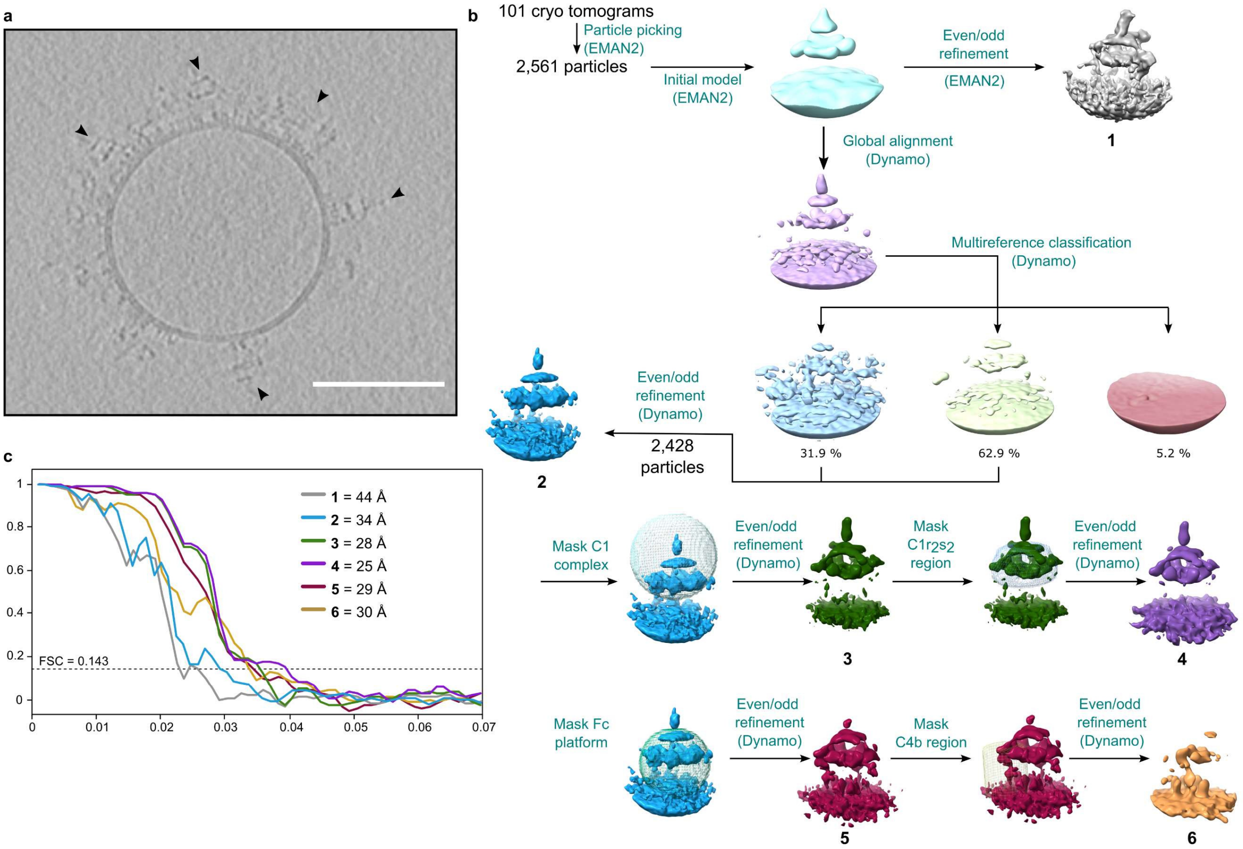

Supplementary Fig. 10. Subtomogram averaging routine of IgG3-C1 complexes and FSC curves. (**a**) Tomographic slice 10 nm thick showing antigenic liposome in the presence of IgG3 and NHS. The scale bar represents 100 nm. Arrowheads indicate C1 complexes bound to antibody platforms. (**b**) Simplified schematic showing the subtomogram averaging routine for C1-complexes bound to IgG3. The final maps described in the text are numbered. (**c**) Fourier shell correlation (FSC) curves for each map. Resolutions reported at FSC = 0.143.

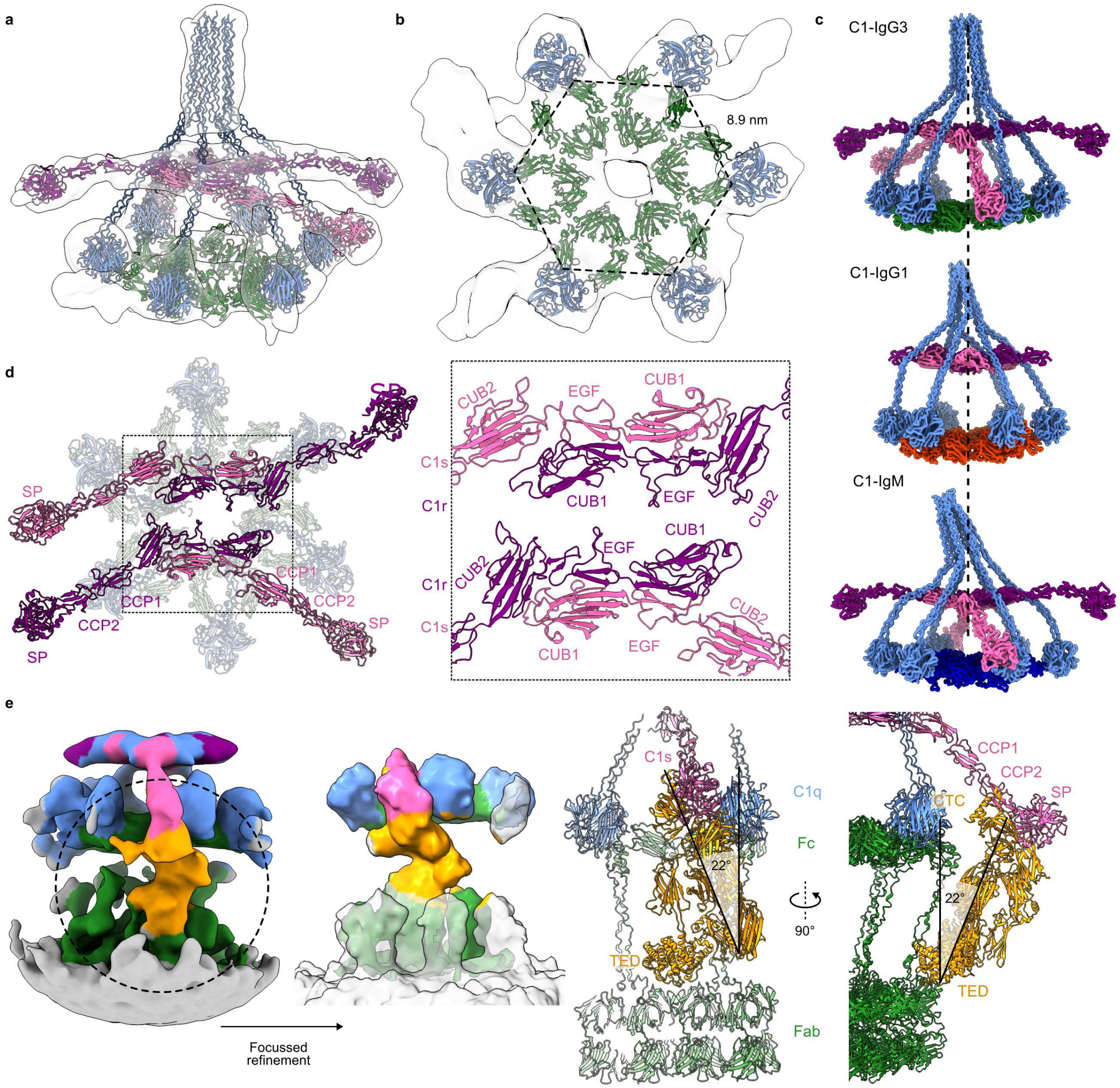

Supplementary Fig. 11. Analysis of IgG3-C1 maps and models.
(**a**) Focussed refinement of IgG3-Fc-C1 region. (**b**) Distances between gC1q headpieces on the hexagonal IgG3-Fc platform. (**c**) Comparison between C1q stalk position. (**d**) Detail of C1r_2s_2 protease platform showing 'complement control protein' (CCP)1, CCP2 and serine proteins (SP) domains. The dashed box is the magnified region showing 'C1r/C1s, Uegf, Bmp1' (CUB)-1, epidermal growth factor (EGF) and CUB2 domains within the C1q complex. (**e**) Focussed refinement of the Fab region revealed density corresponding to C4b. Further focussed refinement around C4b allowed model fitting showing the interaction between C1s (pink) and the C-terminal complement (CTC) domain, with the thioester domain (TED) adjacent to IgG3-Fab domains. C4b is tilted ~22° from vertical.

a

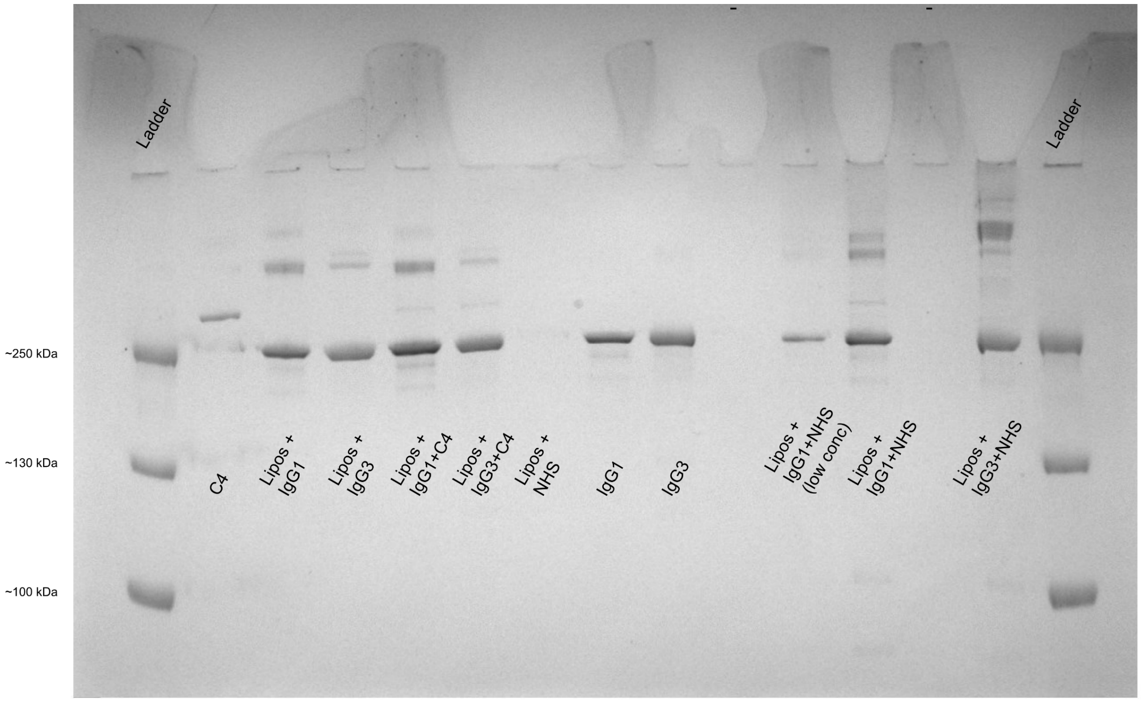
Ladder
C4
Lipos + IgG1
Lipos + IgG3
Lipos + IgG1+C4
Lipos + IgG3+C4
Lipos + NHS
IgG1
IgG3
Lipos + IgG1+NHS (low conc)
Lipos + IgG1+NHS
Lipos + IgG3+NHS
Ladder
~250 kDa
~130 kDa
~100 kDa

b

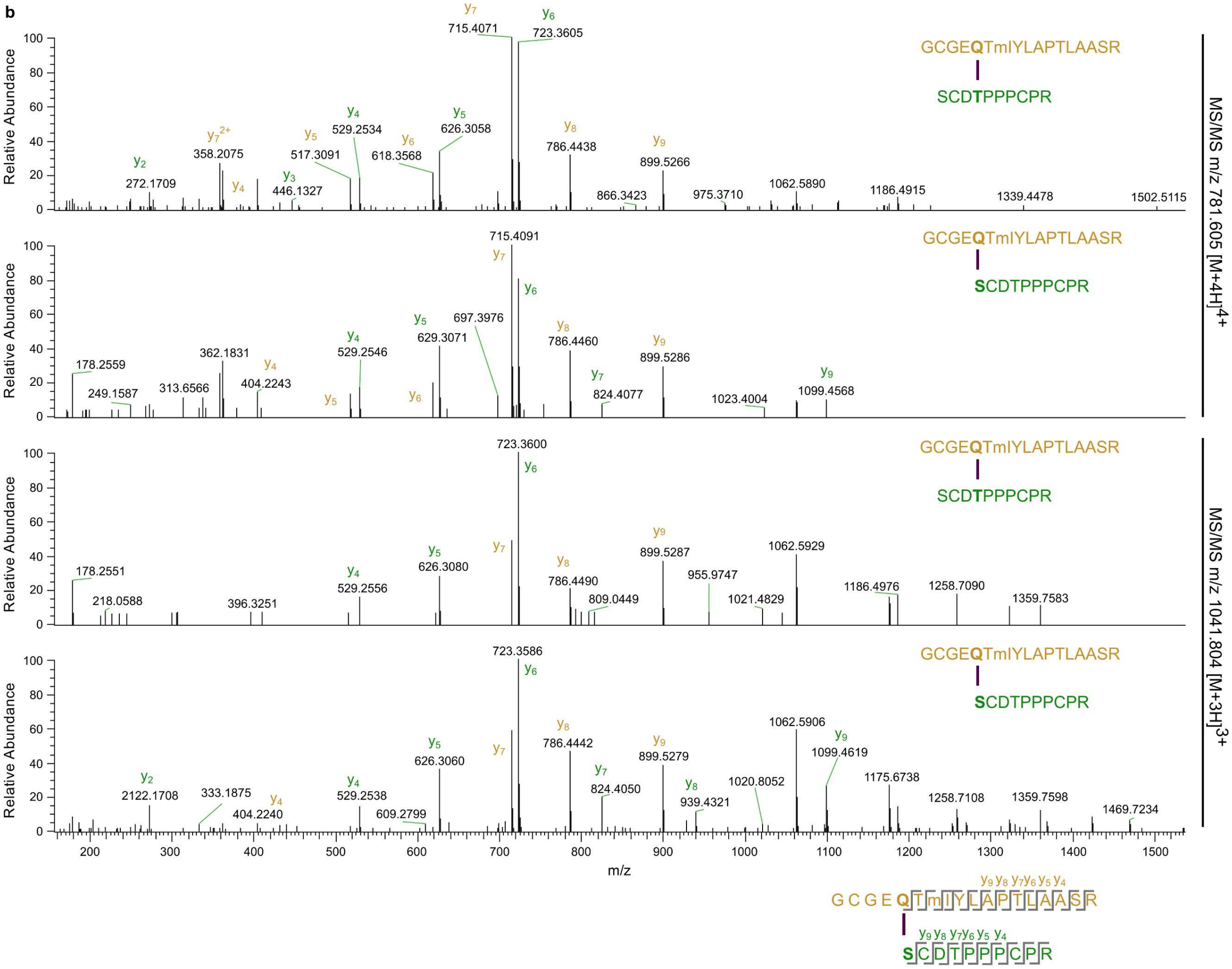
Relative Abundance
m/z
GCGEQTmIYLAPTLAASR
SCDTPPPCPR
MS/MS m/z 781.605 [M+4H]4+
MS/MS m/z 1041.804 [M+3H]3+
715.4071
723.3605
715.4091
723.3600
723.3586
899.5266
786.4438
1062.5890
1062.5929
1062.5906
1099.4568
1099.4619

Supplementary Fig. 12. MS/MS analysis of thioester mediated C4b-IgG3 hinge crosslinked peptides.

(**a**) Example of a Coomassie-stained gel of purified C4, IgG1 and IgG3 mixed in the annotated combinations with liposomes (Lipos) and/or normal human serum (NHS). As a ladder, the PageRuler™ Plus Prestained Protein Ladder, 10 to 250 kDa from Thermo Fisher Scientific was used. Gel was repeated 3 times. (**b**) MS/MS analysis was performed on the quadruply (upper panels) and triply (lower panels) charged species (m/z 781.605 $[M+4H]^{4+}$ and 1041.804 $[M+3H]^{3+}$, respectively). C-terminal fragments (y-ions) are annotated for both the tryptic peptide from C4b (orange, containing the glutamine from the thioester) and the hinge region of IgG3 (green). Based on the fragmentation pattern, crosslinks between the glutamine of C4b and serine at position 238 and threonine at position 241 of IgG3 were observed. Characteristic fragments for the crosslink to serine at position 238 are represented by the y_7-y_9 ions of the **S**CD**T**PPPCPR peptide. The two isomeric crosslinked peptides were also separated in time (Fig. 4d). All cysteines are carbamidomethylated and the methionine (m) in the C4b peptide is oxidised.

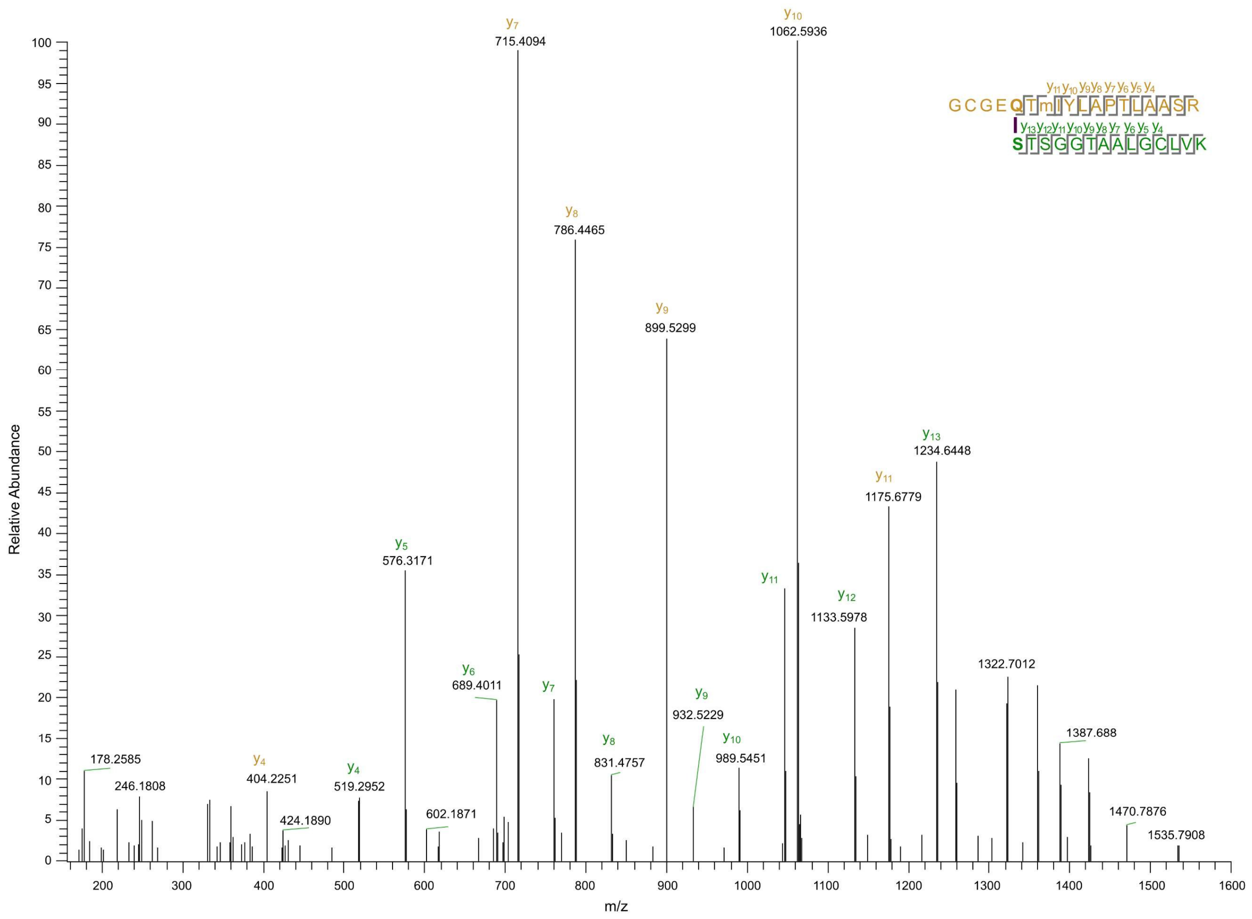

Supplementary Fig. 13. MS/MS analysis of thioester mediated C4b-IgG3 Fab crosslinked peptides. MS/MS analysis was performed on the triply charged species at m/z 1086.864 $[M+3H]^{3+}$. C-terminal fragments (y-ions) are annotated for both the tryptic peptide from the Fab region of IgG3 (green) and the C4b peptide (orange, Q from thioester). Crosslinks between the glutamine of C4b and the serine at position 136 of IgG3 were observed, based on the fragmentation pattern. Characteristic fragments for the crosslink to S136 are represented by y_4-y_{13} ions of the **S**TSGGTAALGCLVK peptide. The crosslinked peptide eluted at 35.37 min (Fig. 4d). All cysteines are carbamidomethylated and the methionine in the C4b peptide is oxidised, indicated as m.

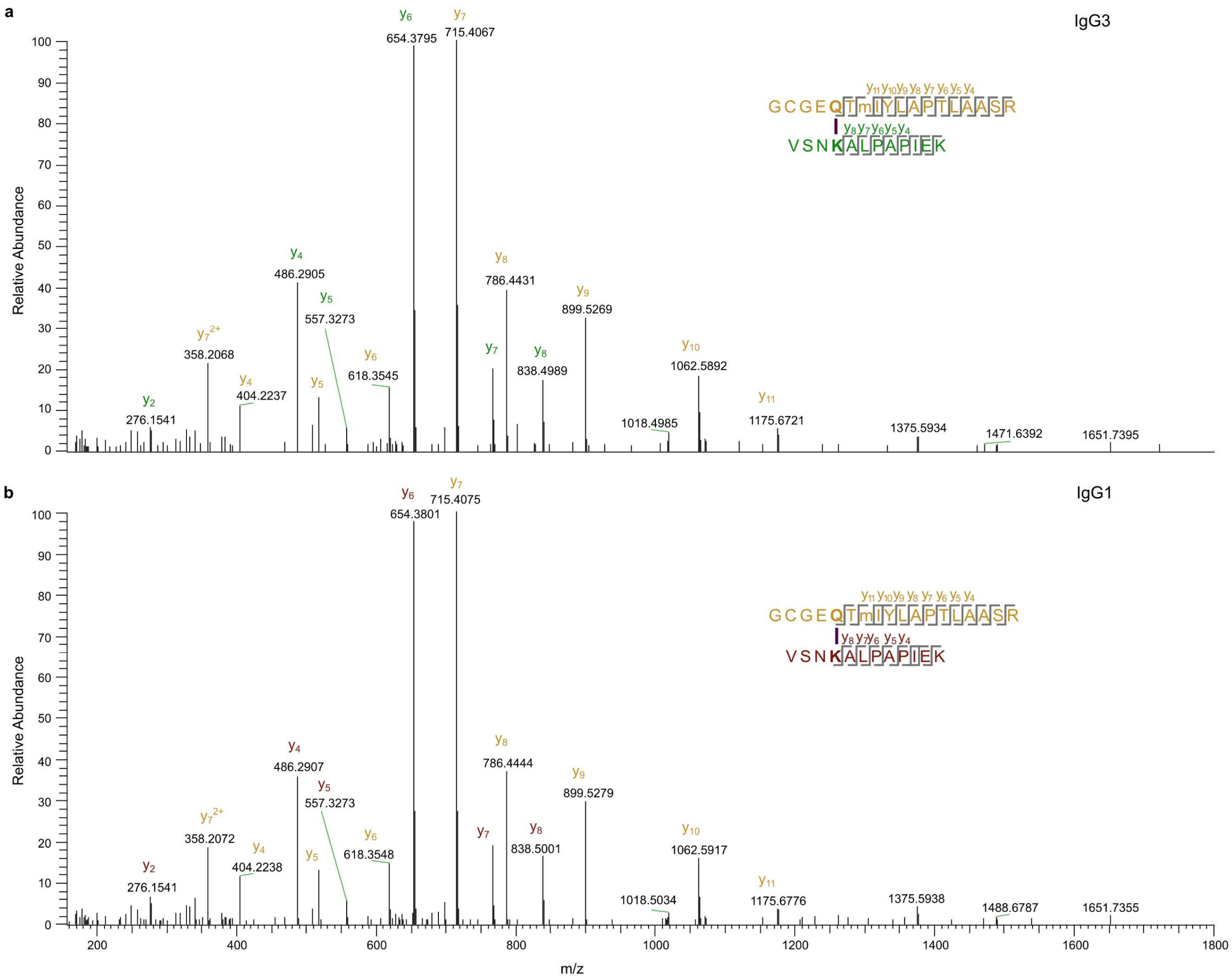

Supplementary Fig. 14. MS/MS analysis of thioester mediated C4b-IgG1 and IgG3 Fc crosslinked peptides.

MS/MS analysis was performed on the quadruply charged species at m/z 801.666 $[M+4H]^{4+}$. C-terminal fragments (y-ions) are annotated for both the tryptic peptide from the Fc region of (**a**) IgG3 (green) or (**b**) IgG1 (red) and the C4b peptide (orange, Q from thioester). Crosslinks between the glutamine of C4b and the lysine at position 328 of IgG1 and lysine at position 375 IgG3 were observed, based on the fragmentation pattern. Characteristic fragments for the crosslink to K328 and K375 for IgG1 and IgG3, respectively, are represented by y_4-y_8 ions of the VSN**K**ALPAPIEK peptide. The crosslinked peptide eluted at 32.22 min and 32.12 min for IgG1 and IgG3, respectively (Fig. 4d). All cysteines are carbamidomethylated and the methionine in the C4b peptide is oxidised, indicated as m.

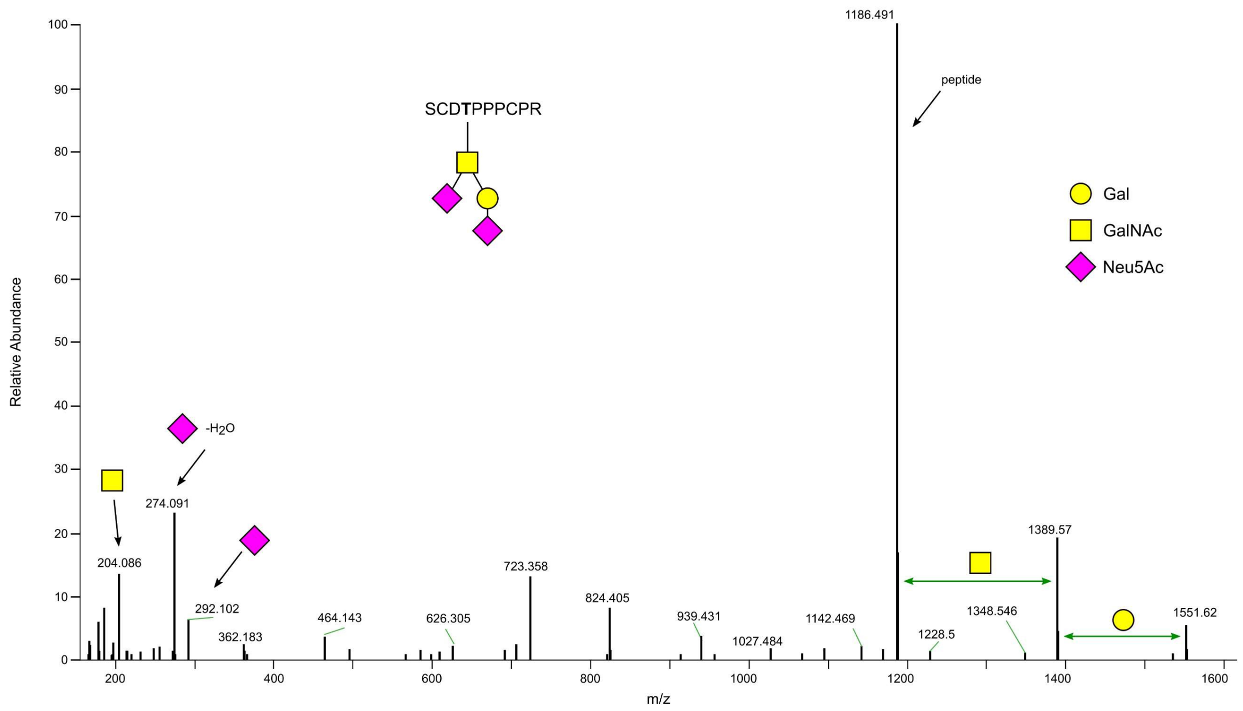

Supplementary Fig. 15. MS/MS analysis of a tryptic O-glycopeptide from IgG3.
Fragmentation spectrum of the tryptic IgG3 peptide SCDTPPPCPR carrying a di-sialyl T-antigen (m/z 1067.4136 [M+2H]2+). All cysteines are carbamidomethylated. Gal (Galactose; yellow circle), GalNAc (N-acetylgalactosamine; yellow square) and Neu5Ac (N-Acetylneuraminic acid; purple diamond). The assignment of the glycan to the threonine residue within this peptide is based on Plomp et al. 12

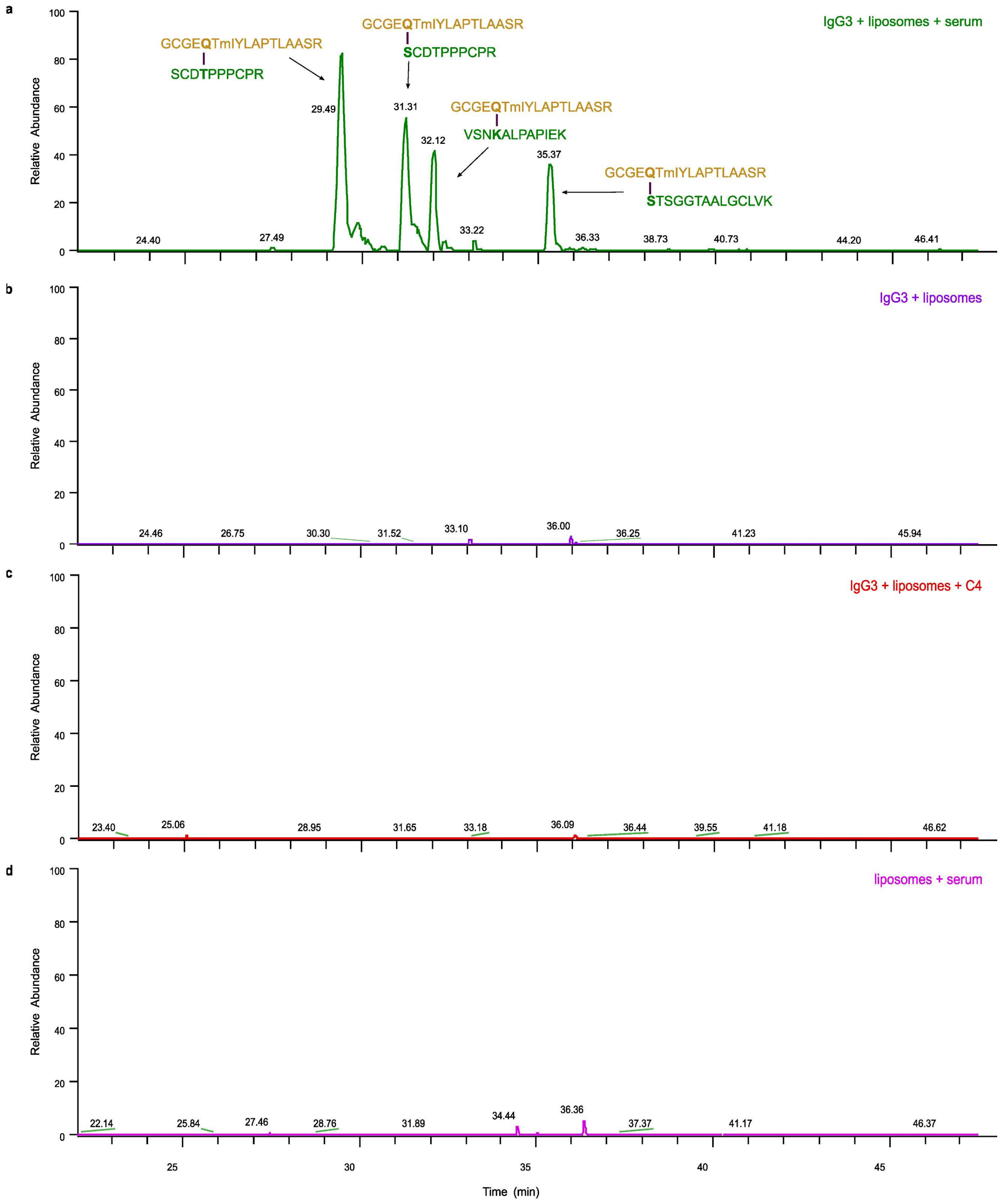

Supplementary Fig. 16. MS characterisation of C4b binding to IgG3 compared to negative controls. (**a**) MS analysis of tryptic peptides form C4b (GCGEQTmIYLAPTLAASR; orange, m = oxidised methionine) crosslinked to IgG3 (green). (**b-d**) No crosslinked peptides to the C4b peptide were detected in any of the negative control. Negative controls include (**b**) IgG3 alone on liposomes (purple), (**c**) IgG3 on liposomes plus C4 (red) and (**d**) liposomes with serum (pink).

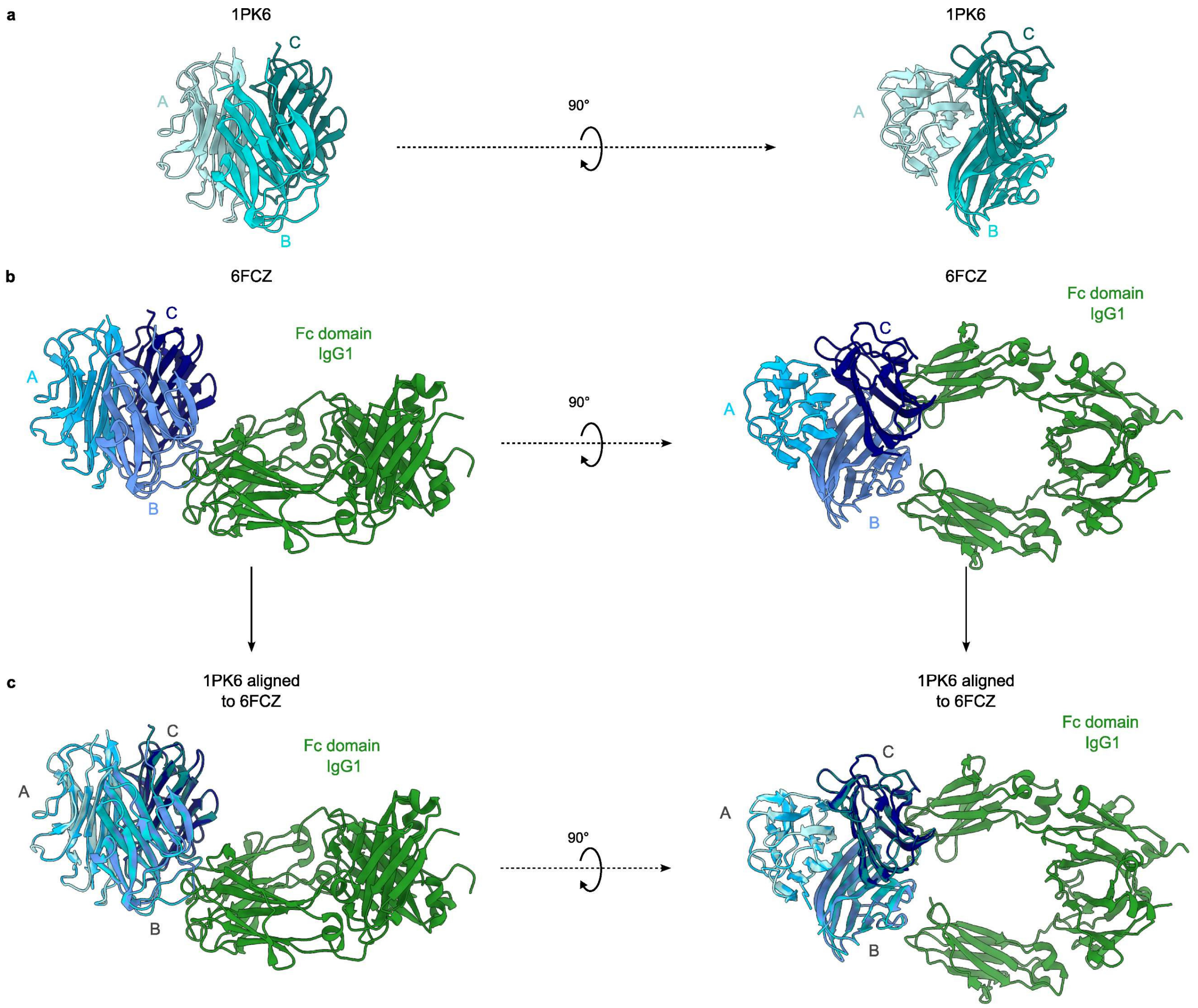

Supplementary Fig. 17. Deposited structures and PDB codes used for the model building of gC1q. (**a**) PDB code 1PK6 showing the globular head of the complement system protein C1q at 1.85 Å (**b**) PDB code 6FCZ showing the model of the gC1q-Fc complex based on an EM map at a resolution of 10.0 Å. (**c**) 6FCZ was used to align 1PK6 binding to the Fc domain of an IgG; 1PK6 was then used to build the model of C1.

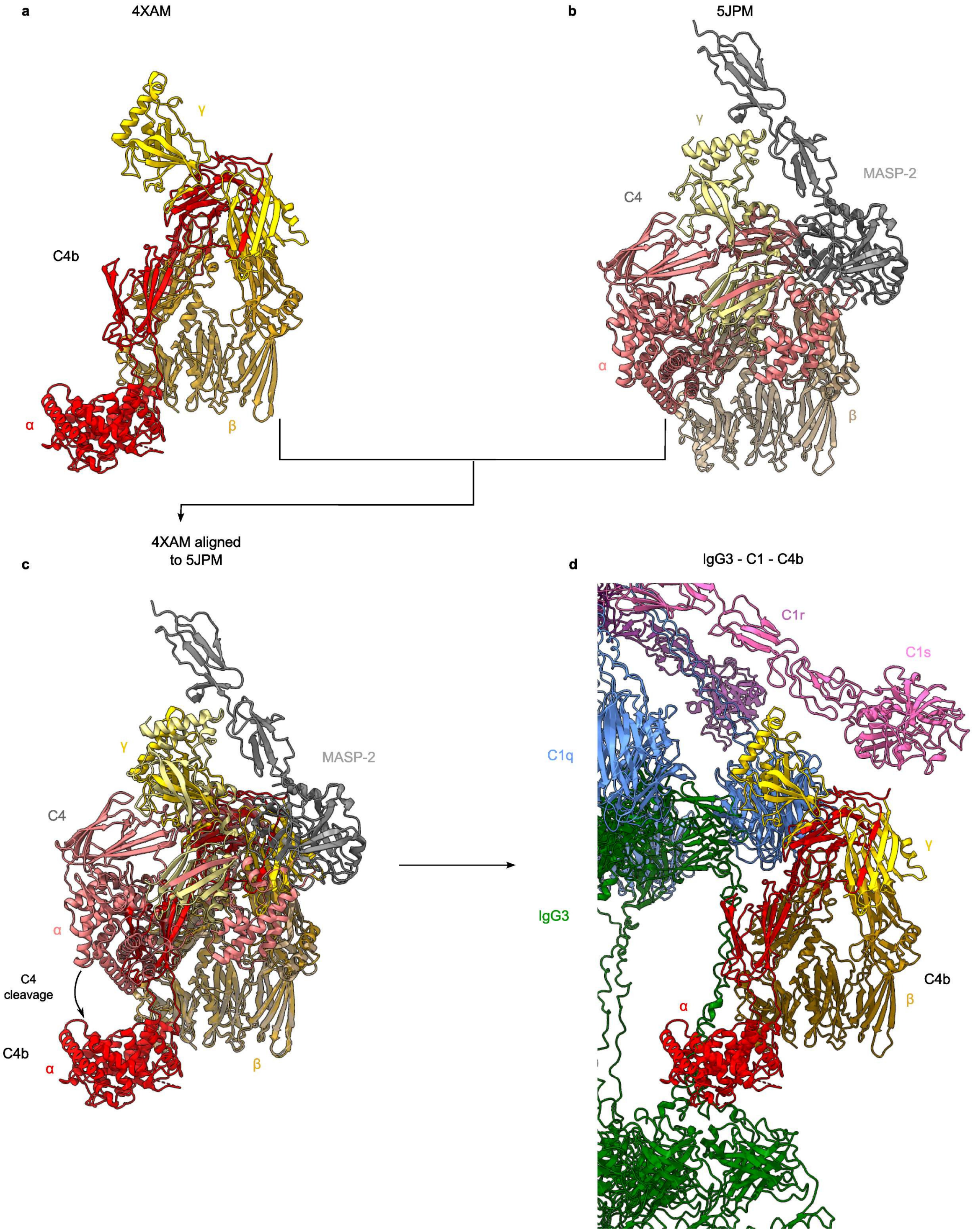

Supplementary Fig. 18. Deposited structures and PDB codes used for model building and alignment of C4b.
(**a**) Structure with PDB code 4XAM showing complement protein C4b at a resolution of 3.5 Å. (**b**) PDB code 5JPM showing the model of the complex of human complement protein C4 with MASP-2 at a resolution of 3.75 Å. (**c**) 5JPM is used to align 4XAM. Whereas 5JPM was only used for alignment, 4XAM was used to build the model shown in this study. (**d**) Model of IgG3-C1-C4b complex, focusing on C4b alignment.

Supplementary Tables

Supplementary Table 1. BLAST comparison of IgG1 and IgG3 constant heavy chain sequences. The upper hinge, disulphide-linked core and lower hinge are highlighted as yellow, cyan and green, respectively. Sites that are linked to C4b are shown in bold and are underlined. IgG3m5 specific amino acids are indicated in red and the highly conserved N297 residue with the N-linked glycan is indicated in magenta. Residues follow Eu numbering. Gaps and "+" indicates non-conservative and conservative mutations, respectively.

```
IgG1   116  SAASTKGPSVFPLAPSSKSTSGGTAALGCLVKDYFPEPVTVSWNSGALTSGVHTFPAVLQ  175
            SAASTKGPSVFPLAP S+STSGGTAALGCLVKDYFPEPVTVSWNSGALTSGVHTFPAVLQ
IgG3   116  SAASTKGPSVFPLAPCSRSTSGGTAALGCLVKDYFPEPVTVSWNSGALTSGVHTFPAVLQ  175

IgG1   176  SSGLYSLSSVVTVPSSSLGTQTYICNVNHKPSNTKVDKRVEPKSC--DKTHTCPPC----  229
            SSGLYSLSSVVTVPSSSLGTQTY CNVNHKPSNTKVDKRVE K+   D THTCP C
IgG3   176  SSGLYSLSSVVTVPSSSLGTQTYTCNVNHKPSNTKVDKRVELKTPLGDTTHTCPRCPEPK  235

IgG1   230  -----------------------------------------PAPELLGGPSVFLFPPKPK  248
                                                     PAPELLGGPSVFLFPPKPK
IgG3   236  SCDTPPPCPRCPEPKSCDTPPPCPRCPEPKSCDTPPPCPRCPAPELLGGPSVFLFPPKPK  295

IgG1   249  DTLMISRTPEVTCVVVDVSHEDPEVKFNWYVDGVEVHNAKTKPREEQYNSTYRVVSVLTV  308
            DTLMISRTPEVTCVVVDVSHEDPEV+F WYVDGVEVHNAKTKPREEQYNST+RVVSVLTV
IgG3   297  DTLMISRTPEVTCVVVDVSHEDPEVQFKWYVDGVEVHNAKTKPREEQYNSTFRVVSVLTV  355

IgG1   309  LHQDWLNGKEYKCKVSNKALPAPIEKTISKAKGQPREPQVYTLPPSREEMTKNQVSLTCL  368
            LHQDWLNGKEYKCKVSNKALPAPIEKTISK KGQPREPQVYTLPPSREEMTKNQVSLTCL
IgG3   356  LHQDWLNGKEYKCKVSNKALPAPIEKTISKTKGQPREPQVYTLPPSREEMTKNQVSLTCL  415

IgG1   369  VKGFYPSDIAVEWESNGQPENNYKTTPPVLDSDGSFFLYSKLTVDKSRWQQGNVFSCSVM  428
            VKGFYPSDIAVEWES+GQPENNY TTPP+LDSDGSFFLYSKLTVDKSRWQQGN+FSCSVM
IgG3   416  VKGFYPSDIAVEWESSGQPENNYNTTPPMLDSDGSFFLYSKLTVDKSRWQQGNIFSCSVM  475

IgG1   429  HEALHNHYTQKSLSLSPGK  447
            HEALHN +TQKSLSLSPGK
IgG3   476  HEALHNRFTQKSLSLSPGK  494
```

Supplementary Table 2. Lengths and sequences of IgG1 and IgG3 hinge regions.
The upper hinge region was measured from the last residue to interact with the κ-light chain to the first disulfide bond between heavy (γ) chains. The core region contains all inter-heavy chain (γ-γ) disulfide bonds. The lower hinge is measured to be between the last γ-γ disulfide bond and the Fc region. See figure below. Sequences are indicated in parentheses.

	IgG1	IgG3
**Upper hinge**	5 amino acids<br>DKTHT	9 amino acids<br>TPLGDTTHT
**Disulphide-linked core**	4 amino acids<br>CPPC	49 amino acids<br>CPRCPEPKSCDTPPPCPRCPEPKSCDTPPPCPRCPEPKSCDTPPPCPRC
**Lower hinge**	9 amino acids<br>PAPELLGGP	9 amino acids<br>PAPELLGGP

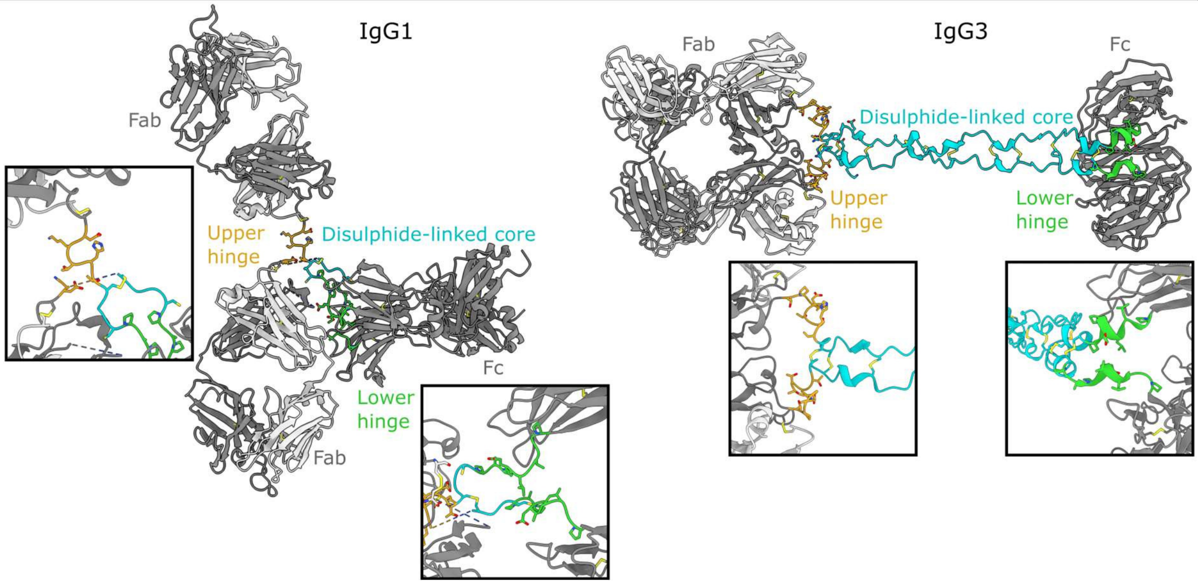

Supplementary Table 3. Parameters for cryoEM data collection and analysis.

**Data collection**	**IgG1**	**IgG1-C1**	**IgG3**		**IgG3-C1-C4b**		
Microscope	Talos Arctica	Talos Arctica	Talos Arctica		Talos Artica		
Voltage (kV)	200	200	200		200		
C2 aperture (µm)	50	50	50		50		
Camera	K3	K3	K3		K3		
Energy filter slit width (eV)	20	20	20		20		
Magnification	49,000	49,000	49,000		49,000		
Pixel size (Å)	1.74	1.74	1.74		1.74		
Tilt range (°)	± 57	± 57	± 57		± 57		
Tilt increment (°)	3	3	3		3		
Tilt scheme	Dose symmetric	Dose symmetric	Dose symmetric		Dose symmetric		
Defocus range (µm)	-3 to -6	-3 to -6	-3 to -6		-3 to -6		
Total electron dose (e/Å^2)	60	60	60		60		
Exposure time per tilt (sec)	0.28	0.3	0.33		0.34		
Movie frames per tilt	10	10	10		10		
Tomograms collected (no.)	12	94	55		104		
Initial subtomograms (no.)	-	-	1,193		2,561		
Final subtomograms (no.)	-	-	571		2,428		
Focussed refinement	-	-	Fc-domain	Fc-domain	general	C1 region	C4b region
Symmetry imposed	-	-	C1	C6	C1	C1	C1
Map resolution (Å) (FSC = 0.143)	-	-	19	14	44	28	30
Map to model resolution (Å) (FSC = 0.5)	-	-	-	24	-	-	-
EMDB	-	-	-	EMD-16227	EMD-16251	EMD-16241	EMD-16250
PDB	-	-	-	8BTB	-	-	-
EMPIAR	-	-	EMPIAR-11406		EMPIAR-11407		

SUPPLEMENTARY REFERENCES

1. Castano-Diez, D., Kudryashev, M., Arheit, M. & Stahlberg, H. Dynamo: a flexible, user-friendly development tool for subtomogram averaging of cryo-EM data in high-performance computing environments. *Journal of Structural Biology* **178**, 139-151 (2012).
2. Gaboriaud, C., Juanhuix, J., Gruez, A., Lacroix, M., Darnault, C., Pignol, D., Verger, D., Fontecilla-Camps, J.C. & Arlaud, G.J. The crystal structure of the globular head of complement protein C1q provides a basis for its versatile recognition properties. *Journal of Biological Chemistry* **278**, 46974-46982 (2003).
3. Ugurlar, D., Howes, S.C., de Kreuk, B.J., Koning, R.I., de Jong, R.N., Beurskens, F.J., Schuurman, J., Koster, A.J., Sharp, T.H., Parren, P. & Gros, P. Structures of C1-IgG1 provide insights into how danger pattern recognition activates complement. *Science* **359**, 794-797 (2018).
4. Gadjeva, M.G., Rouseva, M.M., Zlatarova, A.S., Reid, K.B.M., Kishore, U. & Kojouhorava, M.S. Interaction of Human C1q with IgG and IgM: Revisited. *Biochemistry* **47**, 13093-13102 (2008).
5. Wood, C.W. & Woolfson, D.N. CCBuilder 2.0: Powerful and accessible coiled-coil modeling. *Protein Science* **27**, 103-111 (2018).
6. Bally, I., Ancelet, S., Moriscot, C., Gonnet, F., Mantovani, A., Daniel, R., Schoehn, G., Arlaud, G.J. & Thielens, N.M. Expression of recombinant human complement C1q allows identification of the C1r/C1s-binding sites. *Proceedings of the National Academy of Sciences of the United States of America* **110**, 8650-8655 (2013).
7. Croll, T.I. ISOLDE: a physically realistic environment for model building into low-resolution electron-density maps. *Acta Crystallographica* **D74**, 519-530 (2018).
8. Pettersen, E.F., Goddard, T.D., Huang, C.C., Meng, E.C., Couch, G.S., Croll, T.I., Morris, J.H. & Ferrin, T.E. UCSF ChimeraX: Structure visualization for researchers, educators, and developers. *Protein Science* **30**, 70-82 (2021).
9. Almitairi, J.O.M., Venkatraman Girija, U., Furze, C.M., Simpson-Gray, X., Badakshi, F., Marshall, J.E., Schwaeble, W.J., Mitchell, D.A., Moody, P.C.E. & Wallis, R. Structure of the C1r-C1s interaction of the C1 complex of complement activation. *Proceedings of the National Academy of Sciences of the United States of America* **115**, 768-773 (2018).
10. Budayova-Spano, M., Lacroix, M., Thielens, N.M., Arlaud, G.J., Fontecilla-Camps, J.C. & Gaboriaud, C. The crystal structure of the zymogen catalytic domain of complement protease C1r reveals that a disruptive mechanical stress is required to trigger activation of the C1 complex. *The EMBO journal* **21**, 231-239 (2002).
11. Perry, A.J., Wijeyewickrema, L.C., Wilmann, P.G., Gunzburg, M.J., D'Andrea, L., Irving, J.A., Pang, S.S., Duncan, R.C., Wilce, J.A., Whisstock, J.C. & Pike, R.N. A molecular switch governs the interaction between the human complement protease C1s and its substrate, complement C4. *Journal of Biological Chemistry* **288**, 15821-15829 (2013).
12. Plomp, R., Dekkers, G., Rombouts, Y., Visser, R., Koeleman, C.A.M., Kammeijer, G.S.M., Jansen, B.C., Rispens, T., Hensbergen, P.J., Vidarsson, G. & Wuhrer, M. Hinge-Region O-Glycosylation of Human Immunoglobulin G3 (IgG3). *Molecular & Cellular Proteomics* **14**, 1373-1384 (2015).
