# Supplementary material for: Complement is activated by elevated IgG3 hexameric platforms and deposits C4b onto distinct antibody domains
Source: Nat Commun. 2023 Jul 7;14:4027. doi: 10.1038/s41467-023-39788-5 (PMC10328927; doi:10.1038/s41467-023-39788-5)
Supplement: Supplementary file 3 — Description of Additional Supplementary Files [file 41467_2023_39788_MOESM3_ESM.pdf]

Description of additional supplementary files

File name: Supplementary Data 1

Description: Crosslinked peptides between C4b and IgG1 and 3, respectively, as identified based on LC-MS/MS analysis of tryptic peptides and XlinkX searches. Only crosslinked peptides between C4B and IgG1/3 with a score above 25 are shown.
