# Supplementary material for: Complement is activated by elevated IgG3 hexameric platforms and deposits C4b onto distinct antibody domains
Source: Nat Commun. 2023 Jul 7;14:4027. doi: 10.1038/s41467-023-39788-5 (PMC10328927; doi:10.1038/s41467-023-39788-5)
Supplement: Supplementary file 5 — Reporting Summary [file 41467_2023_39788_MOESM5_ESM.pdf]

nature portfolio

Corresponding author(s): Thomas H. Sharp

Last updated by author(s): Jun 27, 2023

Reporting Summary

Nature Portfolio wishes to improve the reproducibility of the work that we publish. This form provides structure for consistency and transparency in reporting. For further information on Nature Portfolio policies, see our Editorial Policies and the Editorial Policy Checklist.

Statistics

For all statistical analyses, confirm that the following items are present in the figure legend, table legend, main text, or Methods section.

n/a	Confirmed	
☐	☒	The exact sample size (*n*) for each experimental group/condition, given as a discrete number and unit of measurement
☐	☒	A statement on whether measurements were taken from distinct samples or whether the same sample was measured repeatedly
☒	☐	The statistical test(s) used AND whether they are one- or two-sided<br>*Only common tests should be described solely by name; describe more complex techniques in the Methods section.*
☒	☐	A description of all covariates tested
☒	☐	A description of any assumptions or corrections, such as tests of normality and adjustment for multiple comparisons
☐	☒	A full description of the statistical parameters including central tendency (e.g. means) or other basic estimates (e.g. regression coefficient) AND variation (e.g. standard deviation) or associated estimates of uncertainty (e.g. confidence intervals)
☒	☐	For null hypothesis testing, the test statistic (e.g. *F*, *t*, *r*) with confidence intervals, effect sizes, degrees of freedom and *P* value noted<br>*Give P values as exact values whenever suitable.*
☒	☐	For Bayesian analysis, information on the choice of priors and Markov chain Monte Carlo settings
☒	☐	For hierarchical and complex designs, identification of the appropriate level for tests and full reporting of outcomes
☒	☐	Estimates of effect sizes (e.g. Cohen's *d*, Pearson's *r*), indicating how they were calculated

Our web collection on statistics for biologists contains articles on many of the points above.

Software and code

Policy information about availability of computer code

Data collection	SEC data were obtained on a S200increase column (Superdex(TM) 200 Increase 3.2/300; Cytiva)on a ÄKTA(TM) pure micro (former form GE, now Cytiva) setup using the UNICORN version 7.3 software version from Cytiva, measuring wavelengths 230 nm, 260 nm and 280 nm; all measurements were done at 4°C.<br>Biochemical data were measured on a BMG LabTech CLARIOstar (Serial Number: 430-0664) using CLARIOstar software version 5.21 R2 (Firmware Version: 1.15). ELISA data were obtained at an absorbance of 415 nm with a number of cycles of 5 and a cycle time of 84 sec and double orbital shaking of 500 rpm before the first cycle. For the complement activation assay fluorescent increase was measured at an excitation wavelength of 560-20 nm and emission wavelength of 615-40 nm (Dichroic: auto 582.5) with a cycle time of 10 sec and flashes per well and cycles set to 40 and a double orbital shaking of 600 rpm before the first cycle.<br>Tilt series were collected on a Talos Arctica (Thermo Fisher Scientific; 200 kV; 1.74 apix; total dose: 60 e/A2)) equipped K3 direct electron detector and a Bioquantum engery filter (Gatan); microscope using a dose symmetric scheme from 0 to +/- 57 degree with 3 degree increments. FEI Tomography software version 5.5.0 (Thermo Fisher Scientific) was used to generate tilt series.<br>For LC-MS/MS analysis, protein bands were subjected to reduction and alkylation followed by a in-gel trypsin using a Proteineer DP digestion robot (Bruker Daltonics). Peptides were lyophilized and dissolved in 10:0.1 water:formic acid followed by on-line C18 nanoHPLC MS/MS on an Easy nLC 1000 gradient HPLC system (Thermo Fisher Scientific) and an Orbitrap Fusion Lumos Tribrid mass spectrometer (Thermo Fisher Scientific). Samples were injected onto a homemade precolumn (100 µm × 15 mm; Reprosil-Pur C18-AQ 3 µm, Dr. Maisch, Ammerbuch, Germany) and eluted via a homemade analytical nano-HPLC column (30 cm × 50 µm; Reprosil-Pur C18-AQ 3 µm). The nano-HPLC column was drawn to a tip of ?5 µm and acted as the electrospray needle of the MS source. Data were collected in data-dependent MS/MS mode with cycle time of 3 sec. Higher energy collision conditions (HCD) at a normalised collision energy at 32% and recording of MS2 spectrum in the Orbitrap was used for fragmentation.
Data analysis	Biochemical data were analysed in GraphPad Prism version 9.3.1.

Data analysis

Tomograms were reconstructed using IMOD version 4.11. Particles were manually picked in EMAN2.91 and subtomogram averaging was performed in EMAN2.91 and Dynamo version 1.1.532 (used in Matlab version 2021a).
IgG3 Fab domains were modelled using AlphaFold (version 2.2.2) and place on the Fab array in UCSF Chimera (version 1.14).
Collagen fibrils of the C1q arms were modelled in ccbuilder version 2.0.
Model building was performed in Isolde (version 1.5) within UCSF ChimeraX (version 1.5).
N- and O-glycopeptides were identified manually using the Thermo Fisher software XCalibur version 2.2 SP1.48 to display.
Mass spectrometry data were analysed using Proteome Discoverer version 2.5.0.400. XlinkX version 2.5 was used to identify cross-linked-peptides. Score for C4B and IgG1/3 crosslinks were set to 25. MS/MS spectra were interpreted manually.

For manuscripts utilizing custom algorithms or software that are central to the research but not yet described in published literature, software must be made available to editors and reviewers. We strongly encourage code deposition in a community repository (e.g. GitHub). See the Nature Portfolio guidelines for submitting code & software for further information.

Data

Policy information about availability of data

All manuscripts must include a data availability statement. This statement should provide the following information, where applicable:

- Accession codes, unique identifiers, or web links for publicly available datasets
- A description of any restrictions on data availability
- For clinical datasets or third party data, please ensure that the statement adheres to our policy

For Fab-Fab interaction PDB databank was mined and PDB 3WE6, 5TDP, 3HR5 , 4JN2 and 5TDN were observed. Different combinations including a combination of 5TDP, 3HR5 and 5TDN was used. Final Fab array model contained of AlphaFold modelled IgG3-Fab domains placed on a 5TDP array.
For the IgG3-Fc region model (uploaded to PDB - 8BTB), the PDB model with accession number 6D58 was used as a monomer. From this monomer multiple copies were placed in our C6-symmetrised IgG3-Fc map (uploaded to EMDB - EMD-16227) in the same orientation as IgG1-Fc domains were observed in PDB model 1HZH.
For the gC1q, PDB 1K6 was aligned to PDB 6FCZ for orientation purposes. The C1r2s2 proteases of the CUB1-EGF-CUB2 were modelled based the PDB 6F1C. The C1r protease arms (CCP1-CCP2-SP domains) were modelled based on the PDB 1GPZ. The C1s protease arms were modelled based on the PDB 4J1Y.
For C4b, PDB 4XAM was aligned to PDB 5JPM for orientation purpose as it is in agreement with the already published IgM-C1 interacting with C4b (EMD-4945). This orientation of C4b was furthermore fitted in our IgG3-C1-C4b map (uploaded to EMDB - EMD-16241).

CryoEM maps and associated models generated for this study are deposited in the EM database (EMDB) and protein database (PDB) with the following accession codes:
IgG3-Fc hexamer map and model, PDB 8BTB and EMD-16227
IgG3-C1-C4b map, EMD-16241
focussed refinement of C1 region, EMD-16251
focussed refinement of C4b region, EMD-16250

Extracted particles of antigen-bound Ig3 and IgG3-C1 are deposited in the Electron Microscopy Public Image Archive (EMPIAR) with accession codes EMPIAR-11406 and EMPIAR-11407, respectively.
Crosslinked peptides identified via LC-MS/MS analysis and XlinkX searches are supplied as Supplementary Data 1. Mass spectrometry proteomic data have been deposited to the ProteomeXchange Consortium via the Pride partner repository with the dataset identifier PXD039049.

Human research participants

Policy information about studies involving human research participants and Sex and Gender in Research.

Reporting on sex and gender	No research in human research participants was done.
Population characteristics	No research in human research participants was done.
Recruitment	No research in human research participants was done.
Ethics oversight	No research in human research participants was done.

Note that full information on the approval of the study protocol must also be provided in the manuscript.

Field-specific reporting

Please select the one below that is the best fit for your research. If you are not sure, read the appropriate sections before making your selection.

☒ Life sciences ☐ Behavioural & social sciences ☐ Ecological, evolutionary & environmental sciences

For a reference copy of the document with all sections, see nature.com/documents/nr-reporting-summary-flat.pdf

Life sciences study design

All studies must disclose on these points even when the disclosure is negative.

Sample size

Biochemical assays (ELISA and complement activation assay on liposomes) were performed at least times in triplicates, the order of addition

Sample size	was random, and liposomes were freshly produced before each repetition.<br>For cryo-ET data analysis; For cryoET, at least 3 separate grids were prepared independently for each condition described in the text. All tilt series in each dataset were used for reconstruction, and at least 50 tilt series per condition were collected. This number was chosen to ensure a sufficient number of particles were present for each condition. Particles were picked from all reconstructed tomograms manually and all particles were used to build the inital model. Afterwards bad particles were excluded for further analyses.
Data exclusions	No data were excluded from biochemical assays.<br>For cryo-ET data bad classes were excluded. For more information see Material and Methods section as well as supplementary figure S5. This is commonly used based on their consistency with the averaged structures.<br>Threshold for crosslinked peptides was set to a score of 25.
Replication	Biochemical assays were performed at least three times and all data were used for further analysis in GraphPad Prism.<br>Subtomogram average reconstruction are largely deterministic. At least 3 separate grids were prepared independently for each condition and at least 50 tilt series per condition were collected. Subtomogram averaging was performed on > 1000 particles.<br>For LC-MS/MS analysis two independent gels were run and both were successfully analysed.
Randomization	For ELISAs, analytes were added randomly to the plate.<br>For reconstituted systems, analytes were added in the order described in the text by necessity.
Blinding	CryoEM data were split into two even-odd half-datasets and processed semi-independently. The resulting statistics are depicted in the form of Fourier-shell correlation curves, as is standard practice for sub-tomogram averaging and cryoEM image analysis in general. As all reconstructions were done in an automated manner, blinding was not needed.

Reporting for specific materials, systems and methods

We require information from authors about some types of materials, experimental systems and methods used in many studies. Here, indicate whether each material, system or method listed is relevant to your study. If you are not sure if a list item applies to your research, read the appropriate section before selecting a response.

Materials & experimental systems

n/a	Involved in the study	
☐	☒	Antibodies
☐	☒	Eukaryotic cell lines
☒	☐	Palaeontology and archaeology
☒	☐	Animals and other organisms
☒	☐	Clinical data
☒	☐	Dual use research of concern

Methods

n/a	Involved in the study	
☒	☐	ChIP-seq
☒	☐	Flow cytometry
☒	☐	MRI-based neuroimaging

Antibodies

Antibodies used	anti-DNP hIgG1 (based on variable domains mouse monoclonal antibody G2a2; based on isotype variant G1m(f)) - homemade antibody produced by Douwe Dijkstra (for more information see Material and Methods)<br>anti-DNP hIgG3 (based on variable domains mouse monoclonal antibody G2a2; based on isotype variant P01860) - homemade antibody produced by Douwe Dijkstra (for more information see Material and Methods)<br>anti-DNP goat antibody (Bethyl Laboratories, Catalog # A150-117A, polyclonal, Lot # A150-117A-3)<br>α-human C1q rabbit (Dako, Catalog # A0136; polyclonal, Lot # 20083250)<br>α-human C4 goat (Complement Technology, Catalog #A205, polyclonal, Lot # 5c)<br>α-human C5b-9 mouse (Dako, Catalog #M0777, monoclonal Clone aE11, Lot # 41310205)<br>α-goat donkey secondary antibody with HRP (Invitrogen, Catalog # PA1-28664, Lot # VI3087277E)<br>α-rabbit goat secondary antibody with HRP (Dako, Catalog # P0448, Lot # 41255488A)<br>α-mouse goat secondary antibody with HRP (Dako, Catalog # P0447, Lot # 41236467)
Validation	anti-DNP hIgG1 - detailed structural and biophysical analysis in this manuscript<br><br>anti-DNP hIgG3 - detailed structural and biophysical analysis in this manuscript<br><br>anti-DNP goat antibody - https://www.fortislife.com/products/primary-antibodies/goat-anti-dnp-antibody/A150-117A; Acute total body ionizing gamma radiation induces long-term adverse effects and immediate changes in cardiac protein oxidative carbonylation in the rat, Rosen, Kryndushkin, Aryal et al, PLoS One (2020) 15 (6), e0233967 DOI: 10.1371/journal.pone.023396<br><br>α-human C1q rabbit - https://www.labome.com/product/Dako/A0136.html; Bulla R, Tripodo C, Rami D, Ling G, Agostinis C, Guarnotta C, et al. C1q acts in the tumour microenvironment as a cancer-promoting factor independently of complement activation. Nat Commun. 2016;7:10346; Bottermann M, Foss S, Caddy S, Clift D, van Tienen L, Vaysburd M, et al. Complement C4 Prevents Viral Infection through Capsid Inactivation. Cell Host Microbe. 2019;25:617-629.e7;<br>Rosbjerg A, Genster N, Pilely K, Skjoedt M, Stahl G, Garred P. Complementary Roles of the Classical and Lectin Complement Pathways in the Defense against Aspergillus fumigatus. Front Immunol. 2016;7:473<br><br>α-human C4 goat - https://www.complementtech.com/catalog/product/complement-polyclonal-antisera/goat-anti-human-c4/

α-human C5b-9 mouse - https://www.labome.com/product/Dako/M0777.html; Uruha A, Nishikawa A, Tsuburaya R, Hamanaka K, Kuwana M, Watanabe Y, et al. Sarcoplasmic MxA expression: A valuable marker of dermatomyositis. Neurology. 2017;88:493-500; Bahia El Idrissi N, Bosch S, Ramaglia V, Aronica E, Baas F, Troost D. Complement activation at the motor end-plates in amyotrophic lateral sclerosis. J Neuroinflammation. 2016;13:72; Kochounian H, Zhang Z, Spee C, Hinton D, Fong H. Targeting of exon VI-skipping human RGR-opsin to the plasma membrane of pigment epithelium and co-localization with terminal complement complex C5b-9. Mol Vis. 2016;22:213-23; Allenbach Y, Leroux G, SuÃ¡rez Calvet X, Preusse C, Gallardo E, Hervier B, et al. Dermatomyositis With or Without Anti-Melanoma Differentiation-Associated Gene 5 Antibodies: Common Interferon Signature but Distinct NOS2 Expression. Am J Pathol. 2016;186:691-700; Georgiannakis A, Burgoyne T, Lueck K, Futter C, Greenwood J, Moss S. Retinal Pigment Epithelial Cells Mitigate the Effects of Complement Attack by Endocytosis of C5b-9. J Immunol. 2015;195:3382-9; Stratton D, Moore C, Antwi Baffour S, Lange S, Inal J. Microvesicles released constitutively from prostate cancer cells differ biochemically and functionally to stimulated microvesicles released through sublytic C5b-9. Biochem Biophys Res Commun. 2015;460:589-95; Fang C, Manes T, Liu L, Liu K, Qin L, Li G, et al. ZFYVE21 is a complement-induced Rab5 effector that activates non-canonical NF-κB via phosphoinosotide remodeling of endosomes. Nat Commun. 2019;10:2247; Tao J, Lieberman J, Lafayette R, Kambham N. A rare case of Alport syndrome, atypical hemolytic uremic syndrome and Pauci-immune crescentic glomerulonephritis. BMC Nephrol. 2018;19:355; Michailidou I, Naessens D, Hametner S, Guldenaar W, Kooi E, Geurts J, et al. Complement C3 on microglial clusters in multiple sclerosis occur in chronic but not acute disease: Implication for disease pathogenesis. Glia. 2017;65:264-277)

Eukaryotic cell lines

Policy information about cell lines and Sex and Gender in Research

Cell line source(s)	Expi293F™ (catalogue number A1427; Thermo Fisher Scientific, Waltham, Massachusetts, USA) cells were used and transfected using the ExpiFectamine™ 293 Transfection Kit (Thermo Fisher Scientific, Waltham, Massachusetts, USA) following the manufacturer's instructions.
Authentication	We did not do a specific authentication for these cells.
Mycoplasma contamination	We have tested these cells for Mycoplasma and they were negative.
Commonly misidentified lines (See ICLAC register)	N/A
